# Whole-Transcriptome Sequence of Degenerative Meniscus Cells Unveiling Diagnostic Markers and Therapeutic Targets for Osteoarthritis

**DOI:** 10.3389/fgene.2021.754421

**Published:** 2021-10-15

**Authors:** Zongrui Jiang, Xue Du, Xingzhao Wen, Hongyi Li, Anyu Zeng, Hao Sun, Shu Hu, Qing He, Weiming Liao, Zhiqi Zhang

**Affiliations:** ^1^ Department of Joint Surgery, First Affiliated Hospital of Sun Yat-Sen University, Guangzhou, China; ^2^ Guangdong Provincial Key Laboratory of Orthopedics and Traumatology, First Affiliated Hospital of Sun Yat-Sen University, Guangzhou, China; ^3^ Department of Parasitology, Zhongshan School of Medicine, Sun Yat-Sen University, Guangzhou, China; ^4^ State Key Laboratory of Oncology in South China, Collaborative Innovation Center for Cancer Medicine, Department of Musculoskeletal Cancer Surgery, Sun Yat-Sen University Cancer Center, Guangzhou, China; ^5^ Department of Orthopedics, Sun Yat-Sen Memorial Hospital, Guangzhou, China; ^6^ Department of Joint Surgery, Third Affiliated Hospital of Southern Medical Hospital, Guangzhou, China

**Keywords:** meniscus, osteoarthiritis, whole-transcriptome sequence, RNA, co-expression network

## Abstract

Meniscus plays an important role in joint homeostasis. Tear or degeneration of meniscus might facilitate the process of knee osteoarthritis (OA). Hence, to investigate the transcriptome change during meniscus degeneration, we reveal the alterations of messenger RNA (mRNA), microRNA (miRNA), long noncoding RNA (lncRNA), and circular RNA (circRNA) in meniscus during OA by whole-transcriptome sequence. A total of 375 mRNAs, 15 miRNAs, 56 lncRNAs, and 90 circRNAs were significantly altered in the degenerative meniscus treated with interleukin-1β (IL-1β). More importantly, highly specific co-expression RNA (ceRNA) networks regulated by lncRNA LOC107986251-miR-212-5p-SESN3 and hsa_circ_0018069-miR-147b-3p-TJP2 were screened out during IL-induced meniscus degeneration, unveiling potential therapeutic targets for meniscus degeneration during the OA process. Furthermore, lipocalin-2 (LCN2) and RAB27B were identified as potential biomarkers in meniscus degeneration by overlapping three previously constructed databases of OA menisci. LCN2 and RAB27B were both upregulated in osteoarthritic menisci and IL-1β-treated menisci and were highly associated with the severity of OA. This could introduce potential novel molecules into the database of clinical diagnostic biomarkers and possible therapeutic targets for early-stage OA treatment.

## Introduction

Meniscus, a semi-lunar, pale white tissue located between the tibial plateau and femoral condyle, is of great importance to the structure, stability, and biomechanical function of the knee joint (Makris et al., 2011). During attempts to treat common sports injuries such as meniscal tears, previous studies concerning the menisci have been largely focused on applying tissue-engineering techniques to develop qualified meniscus substitutes that possess similar meniscal anatomic structure, biocompatibility, and function (Filardo et al., 2019; Kwon et al., 2019; Shimomura et al., 2019; Zhang et al., 2019). Recently, several studies have discovered that meniscal tears, degeneration, and total meniscectomy are of high relevance with the onset of osteoarthritis (OA), a common chronic disease that mostly occurs in the knee joint (Berthiaume et al., 2005; Hunter et al., 2006; Murphy et al., 2019). The possible “meniscal pathway” to knee OA might be attributed to abnormal biomechanical stress caused by meniscal trauma or dissection (Englund et al., 2012). However, little research has been performed to investigate the specific mechanism underlying meniscal pathology and OA. Therefore, it is necessary to study specific meniscal degenerative mechanisms to cope with the diagnosis and treatment of early OA.

To study the potential regulating mechanism between transcriptional and post-transcriptional modification, bioinformatics has been widely applied. Multiple novel methods have been used to demonstrate the mechanisms responsible for various diseases, for example, with the use of whole-transcriptome sequencing, with which Lei et al. were able to discover the comprehensive circular RNA (circRNA) profile of peripheral blood mononuclear cells in hepatocellular carcinoma (HCC) patients and identified circ_0000798 as a characteristic biomarker for HCC patients (Lei et al., 2019). In orthopedic fields, whole-transcriptome sequencing (Li et al., 2019) and single-cell sequencing (Ji et al., 2019) on human primary OA chondrocytes also assisted with the understanding of the degenerative mechanism of cartilage. Previous studies have also screened out the potential messenger RNA (mRNA), microRNA (miRNA), and long noncoding RNA (lncRNA) in knee cartilage, which might possess cartilage degeneration during OA process (Chen and Chen, 2020; Qi et al., 2020). Recently, with the aid of single-cell sequencing, we were able to identify normal and degenerative meniscus cell types and superficially uncovered that the transition from a meniscus fibrocartilage progenitor (FCP) cell to a meniscus degenerative progenitor cell (DegP) is possibly the key determining factor of the OA meniscus (Sun et al., 2020). However, the molecular mechanism underlying this transition remains unknown.

In this study, we performed whole-transcriptome sequencing on degenerative meniscus with or without interleukin-1β (IL-1β) treatment as inflammatory chemokines, including IL-1β, have been studied as necessary catabolic mediators in OA that are also applicable to the meniscus (McNulty et al., 2013; Cook et al., 2018). Our previous study has also shown that with 48-h IL-1β (5 ng/ml) treatments, the number of FCP cells decreased while DegP increased, possibly causing the transition from FCP to DegP (Sun et al., 2020). Hence, by using IL-1β as an OA inducer in meniscus, we were able to examine the mRNA, miRNA, lncRNA, and circRNA expression profiles in degenerative menisci to identify the characteristics of transcriptional and post-transcriptional differences during the OA degenerative process. Moreover, we overlapped three databases of degenerative menisci to select highly specific biomarkers in the meniscus for diagnosing early-stage OA, including our previous single-cell sequencing on normal menisci and OA degenerative menisci (Sun et al., 2020), whole-transcriptome sequence of IL-1β-abundant menisci, and RNA-seq of control menisci as well as OA degenerative meniscus.

## Materials and Methods

### Isolation and Culture of Human Meniscus Cells

OA meniscus samples were dissected from 10 OA patients who had the indication of total knee arthroplasty (TKA), and the patients who participated in this program offered written informed consent. Healthy meniscus samples were collected from patients who underwent amputation and did not have OA or rheumatoid arthritis. The enrolled criteria included classic clinical history, pains, signs of dyskinesia, and X-ray imaging. The average age and Kellgren-Lawrence grading scores of the patients are listed in Supplemental Table S2. The exclusion criteria and procedures for sample collection and examination were carried out as described in previous studies (Meng et al., 2018). Afterwards, the menisci were cut into slices and digested with 2 mg/ml of collagenase P for 8–12 h and then implanted into medium containing DMEM/Nutrient Mixture F-12 (Gibco Life Technologies, Grand Island, NY, United States ), 5% fetal bovine serum (FBS; Gibco Life Technologies), and 100 IU/ml of penicillin (PS; Gibco Life Technologies). The meniscus cells were cultured in 6-well plates at 37°C in a humidified atmosphere of 5% CO_2_ and 1% oxygen. The cell density was about 1 × 10^7^ per plate.

### Inflammatory Stimulation With Interleukin-1β

For whole-transcriptome sequence, three OA meniscus samples dissected from OA patients were collected and plated for cell culture, named OA004, OA006, and OA008. After the meniscus cells fully adhered to the plate and showed 90% cellular confluency in the 6-well plate, we added 5 ng/ml IL-1β in three wells in each sample, named OA004_IL-1B, OA006_IL-1B, and OA008_IL-1B, while simultaneously added refreshed culture medium as control group (OA004_NC, OA006_NC, and OA008_NC). Three samples were treated with 5 ng/ml of IL-1β to simulate OA inflammatory pathology (OA004_IL-1B, OA006_IL-1B, and OA008_IL-1B), while the other three samples were replaced with refreshed medium instead (OA004_NC, OA006_NC, and OA008_NC). All samples were then cultured at 37°C in a humidified atmosphere of 5% CO_2_ for 48 h.

### Total RNA Extraction

For RNA sequence, four healthy meniscus samples were collected from patients who underwent amputation due to severe femoral fracture who did not have OA or rheumatoid arthritis, and four OA meniscus samples were collected from patients who had the indication of TKA. Total RNA was extracted using TRIzol reagent kit (Invitrogen, Carlsbad, CA, United States) according to the manufacturer’s protocol. We used TRIzol (Invitrogen, Carlsbad, CA, United States) to extract total RNA from each meniscus cell, following the manufacturer’s protocol. The RNA quality was checked by an Agilent 2,200 (Agilent Technologies, Santa Clara, CA, United States) and kept at −80°C, and only samples with an RNA integrity number (RIN) value > 7.0 were used for the cDNA library construction.

### cDNA Library Construction

The cDNA libraries were constructed for each pooled RNA sample using the NEBNext^®^ Ultra™ Directional RNA Library Prep Kit for Illumina (San Diego, CA, United States) according to the manufacturer’s instructions. Generally, the protocol consists of the following steps: depletion of rRNA and fragmented into 150–200 bp using divalent cations at 94°C for 8 min. We further used Dnase I to eradicate contamination after we wiped off ribosome RNA. The cleaved RNA fragments were reverse-transcribed into first-strand cDNA and second-strand cDNA synthesis; fragments were end repaired, A-tailed, and ligated with indexed adapters. Target bands were harvested through AMPure XP Beads (Beckman Coulter, Brea, CA, United States). The products were purified and enriched by PCR to create the final cDNA libraries and quantified by Agilent 2,200. The tagged cDNA libraries were pooled in equal ratio and used for 150-bp paired-end sequencing in a single lane of the Illumina HiSeq X Ten.

The sequencing library of miRNA was prepared from total RNA by using NEBNext Small RNA Library Prep Set for Illumina (NEB) according to the manufacturer’s instructions. Briefly, RNA was ligated with 5′-RNA and 3′-RNA adapters, reversely transcribed into cDNAs, and PCR amplified. The PCR products were size selected and sequenced on HiSeq X Ten platform.

### RNA Sequencing Mapping

Mapping of paired-end reads: Before read mapping, clean reads were obtained from the raw reads by removing the adaptor sequences, reads with >5% ambiguous bases (noted as N), and low-quality reads containing more than 20% of bases with qualities of <20. The clean reads were then aligned to human genome [version: GRCh38 National Center for Biotechnology Information (NCBI)] using the hisat2 (Kim et al., 2015). HTseq (Anders et al., 2015) was used to get gene counts, and RPKM method was used to determine the gene expression. The clean reads of miRNA library were mapped to Human miRNA database (miRBase v22.0) to achieve the miRNA expression.

### Circular RNA Identification and Quantification

The pipeline “acfs,” which was publicly available at https://code.google.com/p/acfs/, was used to identify circRNA in each sample including the following steps (You et al., 2015): Unmapped Reads Collection: BOWTIE2 version 2.2.5 (Langmead and Salzberg, 2012) was used as the mapping method to the respective reference genome [GRCH37.p13 NCBI] utilizing the parameter bowtie2 --end-to-end --sensitive --mm --phred33 --fr --rg-id S13171 --rg SM:S13171 --rg LB:S13171 --rg PL:Illumina -p 8 -X 500 -k 4 -x.)

### Circular RNA Identification

Unmapped reads were collected to identify the circRNA utilizing BWA mem (bwa mem -t 1 -k 16 -T 20): partial alignments of segments within a single read that mapped to 1) regions on the same chromosome and no more than 1 Mb away from each other 2) on the same strand 3) but in reverse order were retained as candidates supporting head-to-tail junction. The strength of potential splicing sites supported by these candidate head-to-tail junction reads was then estimated using MaxEntScan33. The exact junction site was determined by selecting the donor and acceptor sites with the highest splicing strength score. Candidate circRNAs were reported if the head-to-tail junction was supported by at least two reads and the splicing score was greater than or equal to 10.

### Expression Analysis

To estimate the expression of circRNA, we re-aligned all the unmapped reads to the circRNA candidates by using the BWA-mem under the following parameter: bwa mem -t 1 -k 16 -T 20. As for most of the circRNAs, there is no direct evidence for their exact sequence: we filled in the sequence using existing exon annotation. Sequence at the 5′ end was concatenated to the 3′ end to form circular junctions. Reads that mapped to the junction (with an overhang of at least 6 nt) were counted for each candidate.

### Dif-Gene-Finder

We applied EBSeq (Leng et al., 2013) algorithm to filter the differentially expressed genes, after the significant analysis, *p*-value, and false discovery rate (FDR) analysis under the following criteria (Benjamini et al., 2001):

MiRNA under the following criteria: 1) fold change ≥2 or ≤0.5; 2) FDR ≤ 0.05.
mRNA under the following criteria: 1) fold change ≥2 or ≤0.5; 2) FDR ≤ 0.05.
NcRNA under the following criteria: 1) fold change ≥2 or ≤0.5; 2) FDR ≤ 0.05.
CircRNA under the following criteria: 1) fold change ≥2 or ≤0.5; 2) FDR ≤ 0.05.

### Gene Ontology Analysis

Gene Ontology (GO) analysis was performed to facilitate elucidating the biological implications of unique genes in the significant or representative profiles of the target gene of the differentially expressed miRNA in the experiment. We downloaded the GO annotations from NCBI (http://www.ncbi.nlm.nih.gov/), UniProt (http://www.uniprot.org/), and the GO (http://www.geneontology.org/). Fisher’s exact test was applied to identify the significant GO categories, and FDR was used to correct the *p*-values.

### Pathway Analysis

Pathway analysis was used to find out the significant pathway of the differential genes according to Kyoto Encyclopedia of Genes and Genomes (KEGG) database. We turn to Fisher’s exact test to select the significant pathway, and the threshold of significance was defined by *p*-value and FDR.

### GO-Tree

The GO is structured as a directed acyclic graph, and each term has defined relationships to one or more other terms. GO-Tree is built based on the GO directed acyclic graph to provide user-friendly data navigation and visualization. We selected the significant GO-Term (*p*-value < 0.01) in GO analysis based on the up and down differentially expressed genes to construct the GO-Tree to summarize the function affected in the experiment (Zhang et al., 2004).

### Path-Act-Network

KEGG (Ogata et al., 1999) included metabolism, membrane transport, signal transduction, and cell cycle pathways. We picked the genes in enriched biological pathway and using Cytoscape (Shannon et al., 2003) for graphical representations of pathways.

### Target Analysis

We utilized the miRanda (Enright et al., 2003) and the tools for predicting differentially expressed miRNA target on circRNA, lncRNA, and mRNA.

### qRT-PCR and Immunohistochemistry

The RNA extracted from the meniscus cells was reverse-transcribed into cDNA using Super-Script™ III Reverse Transcriptase (Invitrogen). Each primer was designed based on the sequence displayed in the NCBI database. We performed quantitative reverse transcriptase PCR (qRT-PCR) using 2× SYBR Green Master Mix (Arraystar, Rockville, MD, United States ) on an Applied Biosystems (Foster City, CA, United States ) ViiA 7 Real-time PCR System. The final reaction system consisted of 1 µl of cDNA, 3.2 µl of double-distilled water, 0.4 µl of forward and backward primers, and 5 µl of 2× SYBR Green PCR Master Mix. Gene expression levels were measured using the 2^−ΔΔCt^ method. The primer sequences are listed in Supplemental Table S1. In addition, for miRNA validation, total RNA was extracted by miRNeasy Mini Kit (Qiagen, Venlo, Netherlands), and cDNA was synthesized using PrimeScript™ RT Master Mix (Takara, Shiga, Japan). qRT-PCR was performed on a CFX96 system (Bio-Rad, Hercules, CA, United States). GAPDH was used as a housekeeping gene for mRNA, lncRNA, and circRNA, while U6 was applied for miRNA as internal reference genes.

Immunohistochemical analysis was also performed according to previous methods (Sun et al., 2020). For antigen retrieve, sections in 0.1% EDTA were incubated with moderate heat in microwave for 10 min. For staining, sections were treated with 3% normal goat serum for 1 h and incubated with antibodies specific to LCN2 (#26991-1-AP; ProteinTech, Chicago, IL, United States) and RAB27B (#13412-1-AP; ProteinTech).

### Statistical Analysis

Statistical analyses were performed using the Statistical Package for the Social Sciences (SPSS), version 25.0 software (SPSS Inc., Chicago, IL, United States). Data are presented as the mean ± SD of the results of at least three independent experiments. Student’s t-test and the Mann–Whitney *U* test were applied to identify significant differences between groups, where appropriate. Spearman’s rank correlation analysis was used to examine the correlation between two variables (Figure 6D). A *p*-value < 0.05 was considered statistically significant for all tests. In addition, in order to correct the batch effect, RUVseq package from R language was applied for batch correction. Furthermore, the heatmaps and volcano plots were exported by R language Heatmap package 2, and the scatter plots were exported by ggplot2 package.

## Results

### Interleukin-1β Might Facilitate Meniscus Degeneration During Osteoarthritis

**FIGURE 1:**
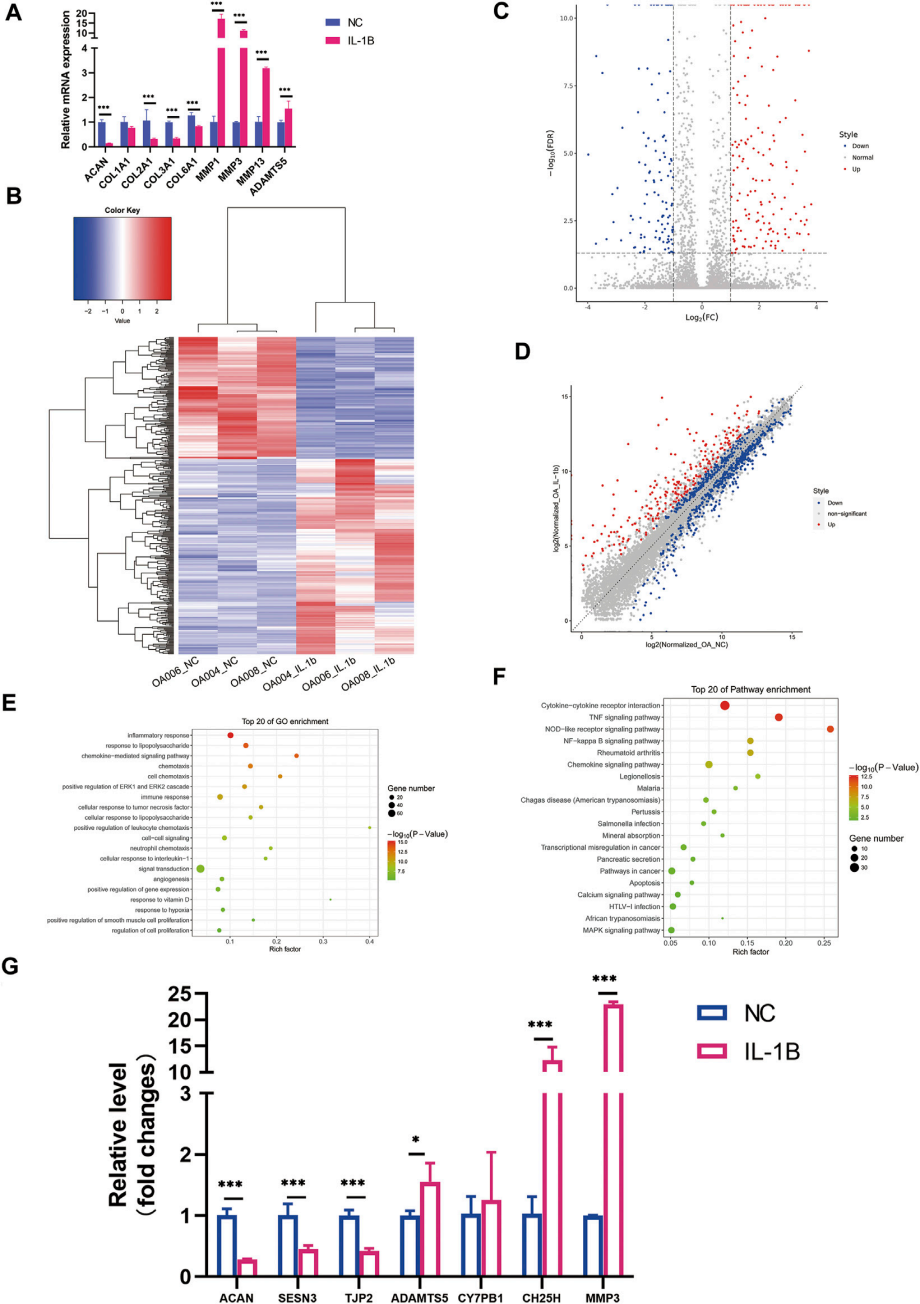
Differential expression profile of messenger RNA (mRNA) between degenerative menisci with and without IL-1β stimulation. **(A)** The expression pattern of meniscus marker genes and inflammatory marker genes in meniscus cells treated with IL-1β (5 ng/ml) as determined by qRT-PCR analysis. GAPDH was used as the internal reference gene for qRT-PCR relative expression. Error bars reveal the standard deviation or the standard error of the data. Student’s t test and Mann–Whitney U test were used to identify significant differences between groups, where appropriate. *p < 0.05, **p < 0.01, ***p < 0.001. **(B)** Hierarchical clustering illustrates distinguished expression difference of mRNA between the two groups and homogeneity between groups. **(C)** Volcano plot of differentially expressed mRNAs. **(D)** Scatter plot of differentially expressed mRNAs. **(E)** The 20 most enriched Gene Ontology (GO) terms for differentially expressed mRNA in degenerative menisci treated with IL-1β. **(F)** The 20 most enriched pathway terms for differentially expressed mRNA in degenerative menisci treated with IL-1β. **(G)** Relative expression levels of selected mRNAs in negative control versus IL-1β-treated osteoarthritis (OA) meniscus. GAPDH was used as the internal reference gene for qRT-PCR relative expression. Error bars reveal the standard deviation or the standard error of the data. Student’s t-test and Mann–Whitney U test were used to identify significant differences between groups, where appropriate. *p < 0.05, **p < 0.01, ***p < 0.001.

To test if IL-1β possesses the effect of meniscus degeneration, we treated menisci with IL-1β (5 ng/ml) for 48 h. As a result, meniscus markers like *COL1A1*, *COL2A1*, *COL3A1*, *COL6A1*, and *ACAN* were significantly downregulated after inflammatory stimulation, while inflammatory markers like *MMP1*, *MMP3*, and *ADAMTS5* were upregulated (Figure 1A). Therefore, we suggest that IL-1β might acquire degenerative effect on meniscus, which is similar with chondrocyte during OA.

### Differential Messenger RNA Expression Profile

A total of 14,800 mRNAs were identified in OA meniscus samples. The hierarchical clustering heatmap, volcano plots, and scatter plots revealed the distinguishable gene expression mapping of each sample (Figures 1B–D). After IL-1β stimulation, 145 mRNAs were significantly downregulated ( $\log_{2}\mathrm{FC}$ < 1, FDR < 0.05), and 230 mRNAs were significantly upregulated ( $\log_{2}\mathrm{FC}$ > 1, FDR < 0.05) compared with those in degenerative meniscus without IL-1β treatment. Among these, aggrecan (*ACAN*) ( $\log_{2}\mathrm{FC}$ = −2.348, FDR = 0) was markedly downregulated, and a disintegrin metallopeptidase with thrombospondin type 1 motif, 5 (*ADAMTS5*) ( $\log_{2}\mathrm{FC}$ = 1.093, FDR = 0.011), cholesterol 25-hydroxylase (*CH25H*) ( $\log_{2}\mathrm{FC}$ = 27.594, FDR = 0), cytochrome P450, family 7, subfamily B, polypeptide 1 (*CYP7B1*) ( $\log_{2}\text{FC}$ = 12.014, FDR = 0), and matrix metalloproteinase 3 (*MMP3*) were significantly upregulated ( $\log_{2}\mathrm{FC}$ = 4.917, FDR = 0.030). As both of them were largely studied in OA cartilage, we further validated the sequencing results using qRT-PCR, and the expression trend was concurrent with the sequencing results (Figure 1G). GO and KEGG pathway analyses were performed to uncover the related functions and signaling pathways of the differentially expressed genes (DEGs). The top 20 enriched GO terms and pathways are listed in Figures 1E,F. DEGs were significantly enriched for inflammatory response (FDR = 5.937E−21) and chemotaxis (FDR = 7.175E−14). Inflammatory signaling pathways such as cytokine–cytokine receptor interactions (FDR = 2.129E−14), TNF (FDR = 2.354E−15), and NOD-like receptor signaling pathways (FDR = 3.248E−15) were remarkably enriched with DEGs upon IL-1β treatment. Interestingly, rheumatic arthritis pathway enrichment was also observed.

### Differentially Expressed microRNA Profile and Its Target Gene Prediction

**FIGURE 2:**
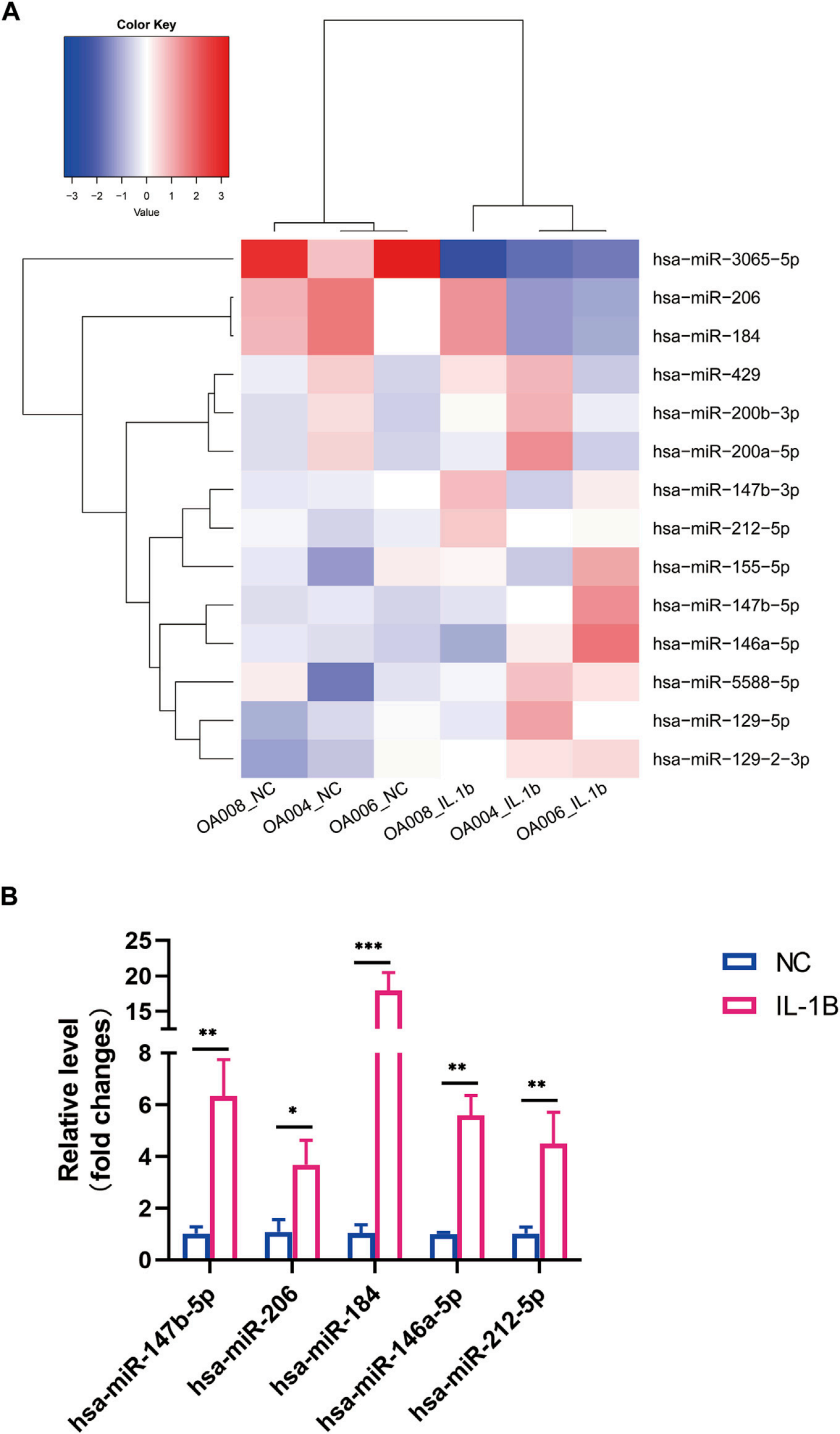
Differential expression profile of microRNA (miRNA) between degenerative menisci with and without IL-1β stimulation. **(A)** Hierarchical clustering illustrates distinguished expression difference of miRNA between the two groups and homogeneity between groups. **(B)** Relative expression level of selected mRNAs in negative control versus IL-1β-treated osteoarthritis (OA) menisci. U6 was used as the internal reference gene for qRT-PCR relative expression. Error bars reveal the standard deviation or the standard error of the data. The statistical methods are described above. *p < 0.05, **p < 0.01, ***p < 0.001.

MiRNA expression was evaluated in OA menisci with or without IL-1β treatment. In total, 1,145 miRNAs were examined, and only 15 differentially expressed microRNA (DEMs) were identified in the hierarchical clustering heatmap ( $\log_{2}\mathrm{FC}$ <1 or >1, FDR < 0.05) (Figure 2A). The most upregulated gene was hsa-miR-147b-5p ( $\log_{2}\mathrm{FC}$ = 3.929, FDR = 3.114E−09). Intriguingly, only one miRNA, hsa-miR-3065-5p, was specifically downregulated by IL-1β stimulation ( $\log_{2}\mathrm{FC}$ = −1.038, FDR = 0.006) (Table 1). qRT-PCR confirmed several upregulated miRNAs expressed in degenerative menisci with or without IL-1β treatment (Figure 2B).

**TABLE 1:** Fourteen differentially expressed miRNAs (OA menisci with vs. without IL-1β treatment; $\;\log_{2}\mathrm{FC}$ < 1, FDR < 0.05).

AccID	log2FC	FoldChange	FDR	Style
hsa-miR-155-5p	1.057178956	2.080858633	1.63276E−06	Up
hsa-miR-3065-5p	−1.038383028	0.486872855	0.006311127	Down
hsa-miR-212-5p	1.773591255	3.419039891	0	Up
hsa-miR-147b-5p	5.526477584	46.09305813	0	Up
hsa-miR-429	2.195607798	4.580826109	0.038617846	Up
hsa-miR-184	5.235364927	37.67054307	0.033117723	Up
hsa-miR-200b-3p	2.341618785	5.068710566	0.00033104	Up
hsa-miR-5588-5p	1.256663751	2.389425441	0.048083509	Up
hsa-miR-146a-5p	3.929859056	15.24071896	3.11491E−09	Up
hsa-miR-129-2-3p	2.013496108	4.037594729	5.29356E−08	Up
hsa-miR-147b-3p	2.652337082	6.286848868	1.32315E−07	Up
hsa-miR-206	5.051225516	33.15663092	0.034566693	Up
hsa-miR-200a-5p	3.063872832	8.362143676	0.046884199	Up
hsa-miR-129-5p	2.068504722	4.194517083	0.044583245	Up

Note. miRNA, microRNA; OA, osteoarthritis; FDR, false discovery rate.

### Expression Profile of Long Noncoding RNAs and Long Noncoding RNA–MicroRNA–Messenger RNA Network Prediction

**FIGURE 3:**
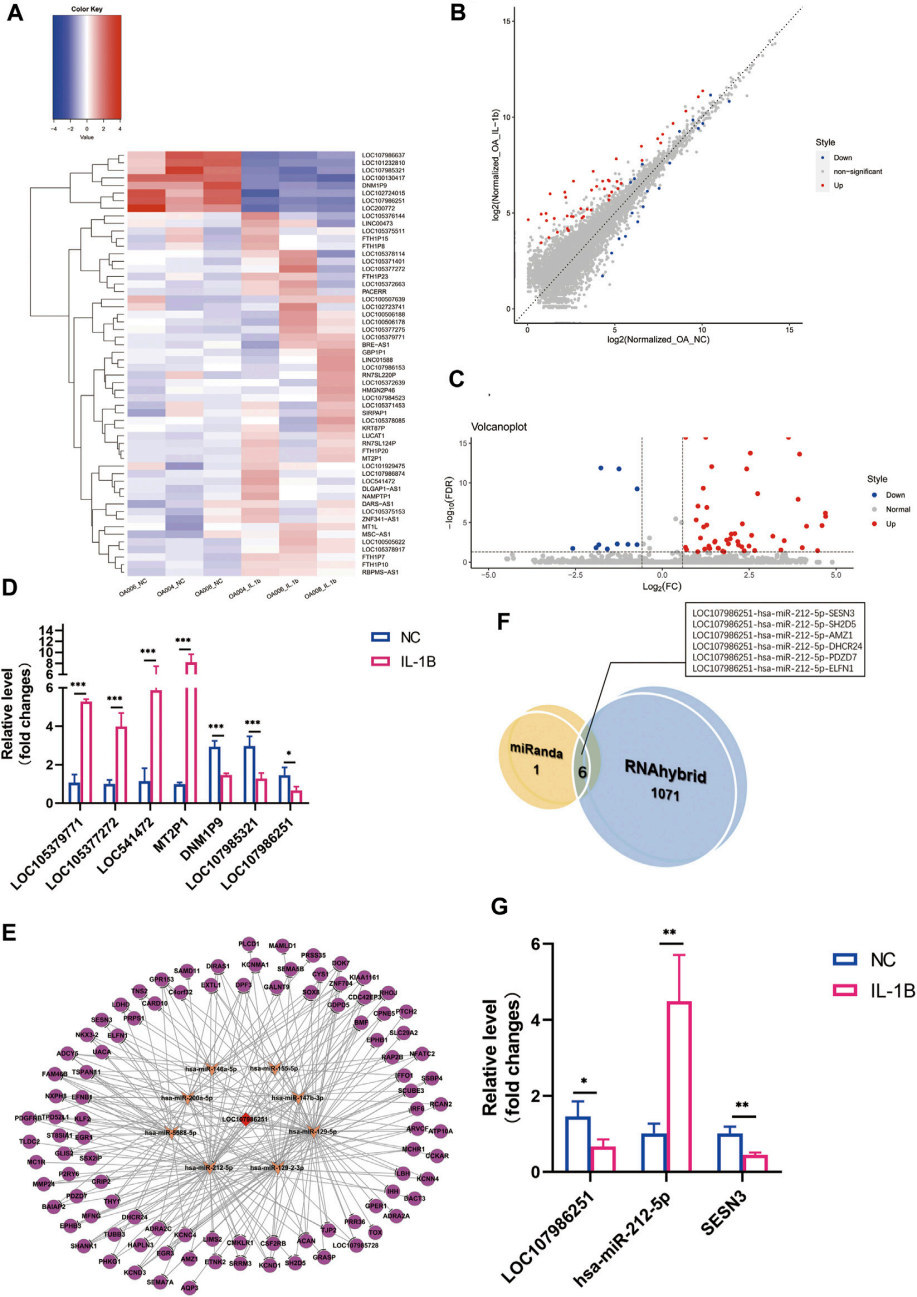
Differential expression profile of long noncoding RNA (lncRNA) and lncRNA LOC107986251 ceRNA network prediction. **(A)** Hierarchical clustering illustrates distinguished expression difference of lncRNA between the two groups and homogeneity between groups. **(B)** Volcano plots of differentially expressed lncRNAs. **(C)** Scatter plots of differentially expressed lncRNAs. **(D)** Relative expression level of selected lncRNAs in negative control versus IL-1β-treated osteoarthritis (OA) menisci. GAPDH was used as the internal reference gene for qRT-PCR relative expression. Error bars reveal the standard deviation or the standard error of the data. The statistical methods are described above. *p < 0.05, **p < 0.01, ***p < 0.001. **(E)** LncRNA LOC107986251 network consists of one lncRNA, eight microRNAs (miRNAs), and 97 mRNAs (RNAhybrid_Energy < −25). The red diamond represents downregulated lncRNA LOC107986251. The orange arrows represent upregulated miRNAs. The purple circles represent suppressed mRNAs. **(F)** Venn diagram of the predicted lncRNA LOC107986251 ceRNA networks by miRanda and RNAhybrid algorithms. **(G)** qRT-PCR validation of LOC107986251, hsa-miR-212-5p, and SESN3 ceRNA regulation pattern upon IL-1β stimulation in degenerative menisci. GAPDH was used as the internal reference gene for qRT-PCR relative expression. Error bars reveal the standard deviation or the standard error of the data. The statistical methods are described above. *p < 0.05, **p < 0.01, ***p < 0.001.

In total, 5,997 lncRNAs were identified in this study, with eight significantly downregulated lncRNAs ( $\log_{2}\text{FC}$ < 1, FDR < 0.05) and 48 upregulated lncRNAs ( $\log_{2}\text{FC}$ > 1, FDR < 0.05) after IL-1β stimulation. LncRNA LOC105379771 ( $\log_{2}\mathrm{FC}$ = 5.482, FDR = 8.689E−05) was the most upregulated lncRNA with nearly a 45-fold change, whereas lncRNA DNM1P9 was the most downregulated ( $\log_{2}\mathrm{FC}$ = −5.002, FDR = 0.0133). Notably, the upregulated lncRNAs were evidently more than the downregulated ones. Hierarchical clustering analysis, volcano plots, and scatter plots revealed distinguishable lncRNA expression profiles between control groups and IL-1β treatment groups (Figures 3A–C), and qRT-PCR validation of four predicted upregulated and three predicted downregulated lncRNAs further confirmed the authenticity (Figure 3D).

Additionally, to determine the potential lncRNA regulation mechanism in the menisci during OA, we performed lncRNA–miRNA–mRNA network analysis using the RNAhybrid algorithm (RNAhybrid_Energy < −25). We identified 1,077 different lncRNA–miRNA–mRNA networks regulated by six different lncRNAs, including LOC100130417, LOC101232810, LOC107986251, LOC200772, and DNM1P9. LncRNA LOC107986251 ( $\log_{2}\mathrm{FC}$ = −1.767, FDR = 1.303E−12) was predicted to have 252 RNA interaction networks in IL-1β-treated degenerative menisci, which possessed the highest amount of RNA interaction networks in the six lncRNAs described earlier (Figure 3E). To discover the most specific and reliable co-expressed RNA (ceRNA) regulatory pathways of lncRNAs, we overlapped the miRanda and RNAhybrid algorithm results (miRanda_Score ≥150, miRanda_Energy < −20, and RNAhybrid_Energy < −25). Consequently, we screened out six ceRNA networks concerning lncRNA LOC107986251 (Figure 3F). A total of 36 different biological processes were identified by GO analysis, and the most enriched were related to regulation of response to oxygen species (FDR = 0.0217) and amyloid precursor protein catabolic processes (FDR = 0.0217) (Supplemental Figure S2A). Only three pathways were confirmed to be enriched in the predicted network during KEGG pathway analysis, in which steroid synthesis (FDR = 0.0177) was the most enriched (Supplemental Figure S2B). Among these, the lncRNA LOC107986251-miR-212-5p-SESN3 network was selected for further qRT-PCR validation in OA menisci with IL-1β treatment, as the downregulation of Sestrin3 (SESN3) in OA cartilage has been described as one of the causes of deficiency in cellular homeostasis, thereby leading to OA. Consequently, validation results were consistent with overlapping prediction (Figure 3G).

### Differential Circular RNA Expression Profile and Circular RNA–MicroRNA–Messenger RNA Network Prediction

**FIGURE 4:**
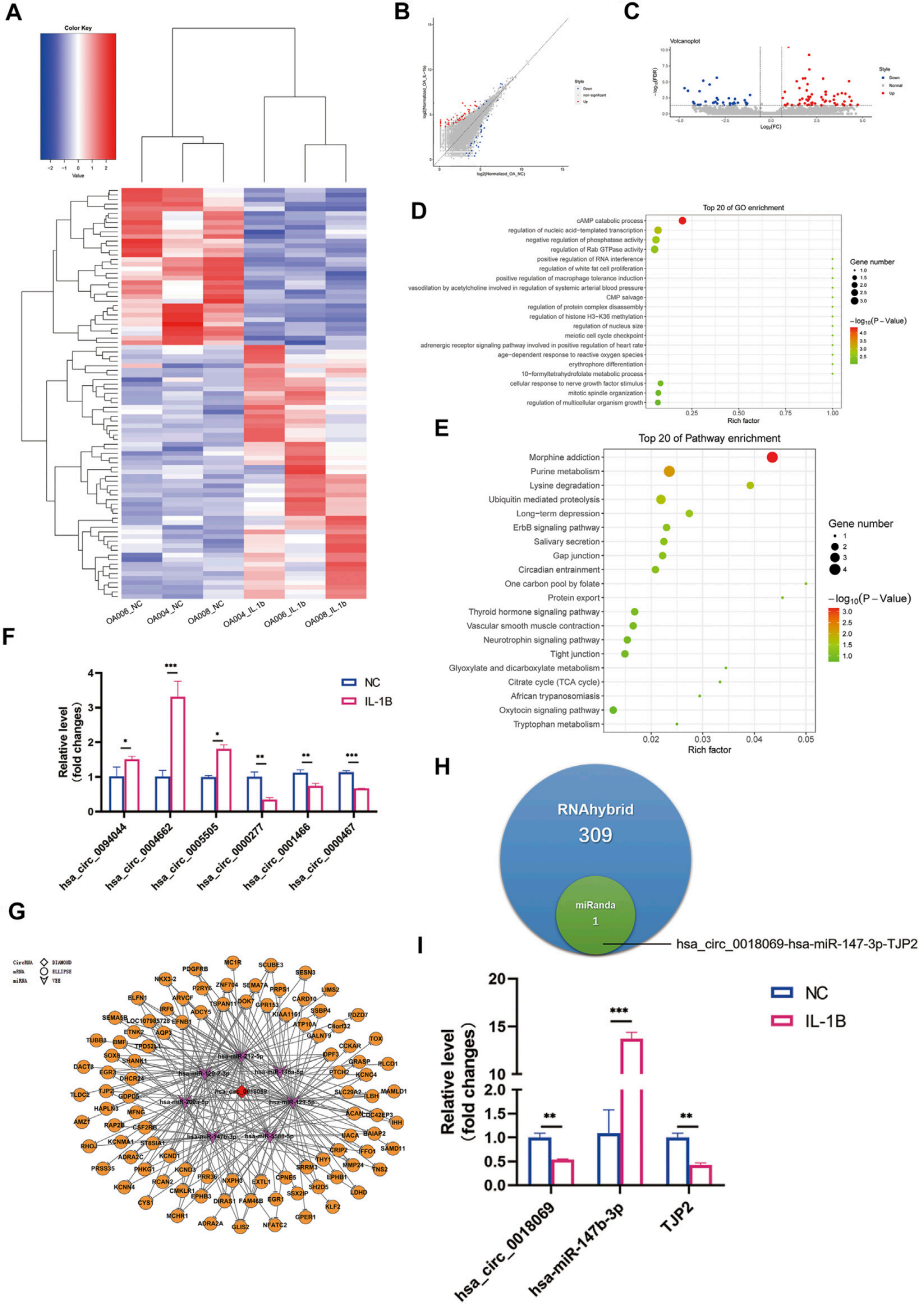
Differential expression profile of circular RNA (circRNA) and potential ceRNA prediction. **(A)** Hierarchical clustering illustrates distinguished expression difference of circRNA between the two groups and homogeneity between groups. **(B)** Volcano plots of differentially expressed circRNAs. **(C)** Scatter plots of differentially expressed circRNAs. **(D)** The 20 most enriched Gene Ontology (GO) terms for the parental genes of differentially expressed circRNA in degenerative menisci treated with IL-1β. **(E)** The 20 most enriched pathway terms for the parental genes of differentially expressed mRNA in degenerative menisci treated with IL-1β. **(F)** Relative expression levels of selected circRNAs in negative control versus IL-1β-treated osteoarthritis (OA) menisci. GAPDH was used as the internal reference gene for qRT-PCR relative expression. Error bars reveal the standard deviation or the standard error of the data. The statistical methods are described above. *p < 0.05, **p < 0.01, ***p < 0.001. **(G)** Hsa_circ_0018069 ceRNA network consists of one circRNA, seven miRNAs, and 97 mRNAs (RNAhybrid_Energy < −25). The red diamond represents downregulated lncRNA LOC107986251. The purple arrows represent upregulated miRNAs. The orange circles represent suppressed mRNAs. **(H)** Venn diagram of the predicted hsa_circ_0018069 ceRNA networks by miRanda and RNAhybrid algorithms. **(I)** qRT-PCR validation of hsa_circ_0018069, hsa-miR-147-3p, and TJP2 ceRNA regulation patterns upon IL-1β stimulation in degenerative menisci. GAPDH was used as the internal reference gene for qRT-PCR relative expression. Error bars reveal the standard deviation or the standard error of the data. The statistical methods are described above. *p < 0.05, **p < 0.01, ***p < 0.001.

A total of 13,715 circRNAs were analyzed concerning differentially expressed circRNA (DECs). The heatmap, volcano plots, and scatter plot results illustrated the distinct circRNA variation between degenerative menisci with and without IL-1β cultivation (Figures 4A–C). A total of 55 circRNAs were significantly upregulated, and 34 circRNAs were significantly downregulated in the IL-1β group compared with those in the no IL-1β group. Further, 73 circRNAs had already been identified in the CircBase database, including 46 upregulated circRNAs and 27 downregulated circRNAs; and qRT-PCR confirmed several circRNA expression patterns (Figure 4F). Among these, hsa_circ_0094044 was the most upregulated ( $\log_{2}\mathrm{FC}$ = 5.926, FDR = 5.288E−07), whereas hsa_circ_0000277 expression was the most evidently suppressed ( $\log_{2}\mathrm{FC}$ = −4.716, FDR = 9.706E−05). Additionally, 17 circRNAs were not marked in the CircBase database, suggesting several novel circRNAs for further investigation. GO analysis indicated the top 20 highly enriched GO terms and proposed that the parental gene of DECs were largely enriched for cAMP catabolic process (FDR = 0.0900), regulation of nucleic acid-templated transcription (FDR = 0.0900), negative regulation of phosphatase activity (FDR = 0.1159), and Rab GTPase activity (FDR = 0.1158) (Figure 4D). Pathway analysis also revealed the 20 most enriched pathways (Figure 4E). The downregulated transcripts were notably enriched in the case of morphine addiction. For the upregulated transcripts, lysine degradation (FDR = 0.2918) was remarkably enriched. In addition, purine metabolism (FDR = 0.2918), which is highly associated with the pathophysiology of urarthritis, was also prominently enriched with DECs upon IL-1β stimulation.

By merely applying the RNAhybrid algorithm (RNAhybrid_Energy < −25), 1,024 circRNA–miRNA–mRNA networks were predicted to be involved in IL-1β-stimulated degenerative menisci concerning 13 DECs; and hsa_circ_0018069 ( $\log_{2}\mathrm{FC}$ = −3.030, FDR = 0.0135) was established to be involved in the regulation of 246 ceRNA networks (Figure 4G), which possesses the highest amount of ceRNA relation networks. Only one ceRNA regulatory pathway, hsa_circ_0018069-miR-147b-3p-TJP2 network, was screened out by overlapping the miRanda and RNAhybrid algorithm results (miRanda_Score ≥150, miRanda_Energy < −20, and RNAhybrid_Energy < −25) (Figure 4H). qRT-PCR also confirmed the expression pattern in degenerative menisci with IL-1β stimulation (Figure 4I). Furthermore, GO and KEGG pathway analyses showed that the ceRNA network was highly enriched in biological pathway like the regulation of membrane permeability (FDR = 0.0019) (Supplemental Figures S3A,B).

### qRT-PCR Validations in Normal and Degenerative Menisci and Screening for Potential Diagnostic Messenger RNA Biomarkers in the Menisci During Early-Stage Osteoarthritis

**FIGURE 5:**
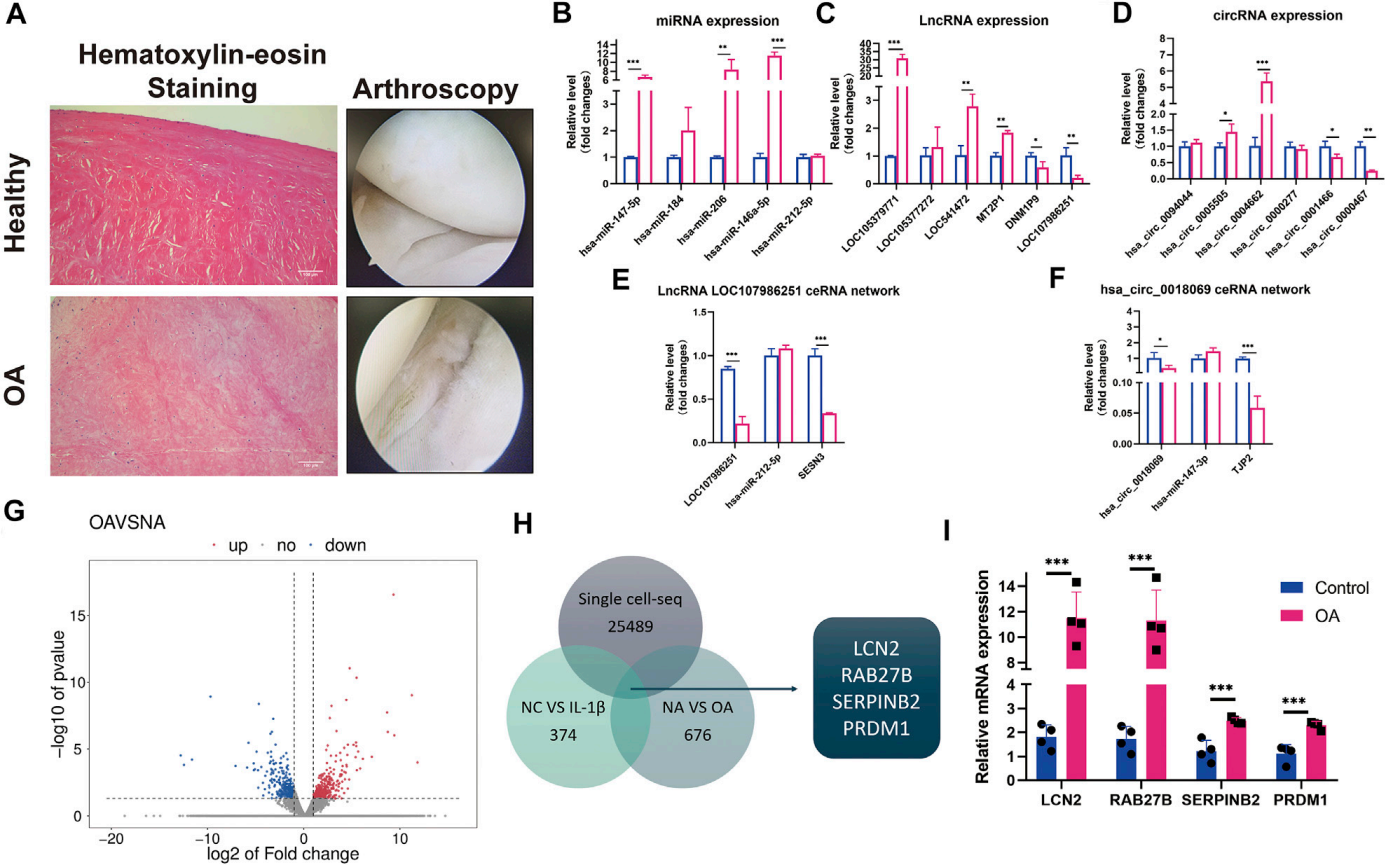
qRT-PCR certification on control and degenerative meniscus and the selection of potential osteoarthritis (OA) biomarkers in meniscus. **(A)** Hematoxylin–eosin staining and arthroscopy of the meniscus from normal and degenerative OA meniscus. **(B)** Relative expression levels of selected differentially expressed microRNAs (DEMs) in normal versus degenerative meniscus. U6 was used as the internal reference gene for qRT-PCR relative expression. Error bars reveal the standard deviation or the standard error of the data. The statistical methods are described above. *p < 0.05, **p < 0.01, ***p < 0.001. **(C)** Relative expression levels of selected differentially expressed circRNA (DECs) in normal versus degenerative meniscus. GAPDH was used as the internal reference gene for qRT-PCR relative expression. Error bars reveal the standard deviation or the standard error of the data. The statistical methods are described above. *p < 0.05, **p < 0.01, ***p < 0.001. **(D)** Relative expression levels of selected differentially expressed lncRNAs (DELs) in normal versus degenerative meniscus. GAPDH was used as the internal reference gene for qRT-PCR relative expression. Error bars reveal the standard deviation or the standard error of the data. The statistical methods are described above. *p < 0.05, **p < 0.01, ***p < 0.001. **(E)** Relative expression patterns of lncRNA LOC107986251hsa_circ_0018069 in normal versus degenerative meniscus. GAPDH was used as the internal reference gene for qRT-PCR relative expression. Error bars reveal the standard deviation or the standard error of the data. The statistical methods are described above. *p < 0.05, **p < 0.01, ***p < 0.001. **(F)** Relative expression patterns of hsa_circ_0018069 in normal versus degenerative meniscus. GAPDH was used as the internal reference gene for qRT-PCR relative expression. Error bars reveal the standard deviation or the standard error of the data. The statistical methods are described above. *p < 0.05, **p < 0.01, ***p < 0.001. **(G)** The scatter plots of differentially expressed mRNAs between normal and degenerative menisci. **(H)** The Venn diagram of single-cell sequence data of normal meniscus versus degenerative meniscus, whole-transcriptome sequence data of control versus IL-1βstimulated OA degenerative meniscus, and RNA sequence data of normal meniscus versus OA degenerative meniscus. **(I)** qRT-PCR confirmation of the screening mRNA (*LCN2*, *RAB27B*, *PRDM1*, and *SERPINB2*). GAPDH was used as the internal reference gene for qRT-PCR relative expression. Error bars reveal the standard deviation or the standard error of the data. The statistical methods are described above. *p < 0.05, **p < 0.01, ***p < 0.001.

To further confirm the results of whole-transcriptome sequencing, we selected previous qRT-PCR-verified DEMs, differentially expressed lncRNAs (DELs), and DECs to validate their expression patterns between normal and degenerative menisci by qRT-PCR (Figure 5A). Consequently, three out of five miRNAs, five out of six lncRNAs, and four out of six circRNAs were shown to have concurrent expression trends with the sequencing results and qRT-PCR validation between the OA menisci with and without IL-1β stimulation (Figures 5B–D). In addition, the lncRNA LOC107986251-miR-212-5p-SESN3 network and hsa_circ_0018069-miR-147b-3p-TJP2 network were also confirmed (Figures 5E,F). Interestingly, miR-147-3p and miR-212-5p expression were not significantly altered between normal and degenerative menisci, unlike in IL-1β-treated groups. This phenomenon may be largely attributed to stronger and immediate exogenous chemokine inflammatory-stimulated effects on fragile degenerative meniscus tissue. Inflammatory stimulation of endogenous chronic chemokines might not be strong enough for the significant alterations of miR-147-3p, miR-212-5p, and other unchanged ncRNAs.

Simultaneously, we performed RNA-seq on four healthy control menisci and four degenerative menisci in order to select potential diagnostic biomarkers for early-stage OA (Figure 5G). *LCN2*, *RAB27B*, *SERPINB2*, and *PRDM1* were screened out by overlapping previously constructed single-cell sequencing data on normal and OA menisci (Sun et al., 2020), and whole-transcriptome sequence data on IL-stimulated meniscus (Figure 5H).

### *LCN2* and *RAB27B* Might Act as Biomarkers in Meniscus for OA Severity Predictors and Early OA Diagnosis

**FIGURE 6:**
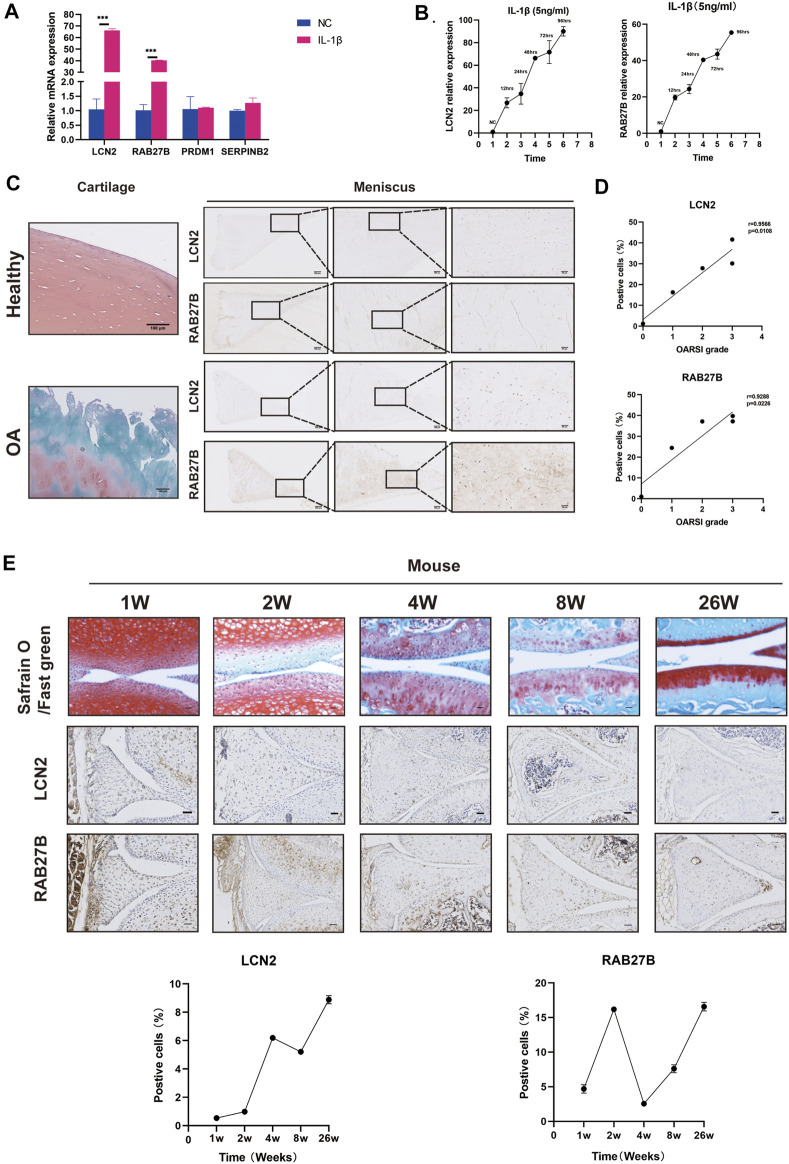
*LCN2* and *RAB27B* might act as biomarkers in the meniscus for early osteoarthritis (OA) diagnosis. **(A)** Relative expression levels of *LCN2*, *RAB27B*, *PRDM1*, and *SERPINB2* in IL-1β-treated menisci. GAPDH was used as the internal reference gene for qRT-PCR relative expression. Error bars reveal the standard deviation or the standard error of the data. The statistical methods are described above. *p < 0.05, **p < 0.01, ***p < 0.001. **(B)** Relative expression levels of *LCN2* and *RAB27B* in menisci treated with time-dependent IL-1β stimulation (0, 12, 24, 48, 72, and 96 h). GAPDH was used as the internal reference gene for qRT-PCR relative expression. Error bars reveal the standard deviation or the standard error of the data. **(C)** Safranin O/Fast Green staining of patients’ knee cartilage and immunohistochemical (IHC) staining of patients’ menisci with antibody against LCN2 and RAB27B. **(D)** Correlation coefficient between LCN2 and RAB27B expression quantified by IHC positive cell percentage and OARSI score in patients’ samples (*n* = 5). **(E)** Safranin O and IHC staining of LCN2 and RAB27B in mice knee at 1, 2, 4, 8, and 26 weeks and quantification of positive cells (*n* = 3). Error bars reveal the standard deviation or the standard error of the data. Scale bar, 50 μm.

We further examined whether meniscus-specific *LCN2* and *RAB27B* possess the potential of predicting OA severity. qRT-PCR confirmed *LCN2* and *RAB27B* expression patterns, both of which show significant upregulation in OA degenerative menisci, while also time-dependently upregulated in inflammatory chemokine-stimulated menisci (Figures 5I, 6A,B). Interestingly, *LCN2* and *RAB27B* expression showed robust correlation with patients’ OA severity based on OARSI Osteoarthritis Cartilage Histopathology Assessment System (Waldstein et al., 2016; Figure 6C). *LCN2* and *RAB27B* were also examined in spontaneous aging C57BL/6J mouse model to validate if meniscus-specific *LCN2* and *RAB27B* could act as biomarkers for early-stage OA. Both of them were discovered to be significantly upregulated at the age of 26 weeks, which is approximately 40 years old in human lifespan (Figure 6D). This suggests that *LCN2* and *RAB27B* might be potential diagnostic biomarkers in meniscus for OA severity prediction and early-stage OA diagnosis.

## Discussion

Whole-transcriptome sequencing is a novel bioinformatics analysis method to test the differential expression levels of mRNA, miRNA, lncRNA, and circRNA between normal and pathological tissues. This technique has already been widely applied in the field of oncology (Zheng et al., 2016). A recent study illustrated the comprehensive transcriptome map of normal and OA cartilage and identified four DELs and six DEGs targeted by lncRNAs during OA (Li et al., 2019). Potential OA-associated genes, pathways, competing endogenous RNA networks, and co-expression networks in knee cartilage were further identified in lately studies, thereby offering a better understanding of OA mechanism (Chen and Chen, 2020; Qi et al., 2020). However, a comprehensive analysis of the expression patterns of mRNA, miRNA, lncRNA, and circRNA in OA meniscus, another important knee joint anatomic structure, remains unknown. A previous study had already described that IL-1β stimulation on chondrocytes could act as an *in vitro* model for OA (Kapoor et al., 2011). Simultaneously, IL-1β performed similar effects on menisci in our study. Therefore, we systematically analyzed the expression profile in degenerative menisci obtained from patients with last-stage OA with or without IL-1β treatment. As a result, we identified 14,800 genes, 1,145 miRNAs, 5,997 lncRNAs, and 13,715 circRNAs. Among these, 375 mRNAs, 15 miRNAs, 56 lncRNAs, and 56 circRNAs were significantly modified subsequent to IL-1β treatment. Following principal component analysis (PCA), we have discovered that sample OA006_NC exhibited high heterogeneity as compared with OA004_NC and OA008_NC (Supplemental Figure S1). This phenomenon might contribute to slight influence on the following sequence results, and we will discuss it in our limitations.

A total of 375 DEGs were examined, and upregulated genes were remarkably more pronounced than downregulated genes. With this, our study confirmed several DEGs that were previously discussed in previous research on OA cartilage, including *MMP3* (Shi et al., 2016), superoxide dismutase 2 (*SOD2*) (Fu et al., 2016), *ADAMTS5* (Mokuda et al., 2019), *CH25H*, cytochrome P450, family 7, subfamily B, polypeptide 1 (*CYP7B1*) (Choi et al., 2019), and bone morphogenetic protein 2 (*BMP2*) (Blaney Davidson et al., 2015). Nonetheless, several genes that were found to be differentially expressed in degenerative menisci, such as *COL1A1* and *COL10A1* (Brophy et al., 2017), were not significantly altered in our study. The lack of sample abundance might contribute to this phenomenon. In terms of GO and KEGG pathway analyses, most enriched genes were highly associated with biological processes implicated in inflammation, such as inflammatory response, chemokine-mediated signaling pathways, chemotaxis, and response to lipopolysaccharide, potentially contributing to meniscus inflammation during the degenerative process. Based on these data, it is possible that IL-1β might contribute to the initiation of general chronic knee joint inflammation within menisci.

The attempt to test the DEMs permitted the discovery of the possible co-expression RNA (ceRNA) regulation networks of lncRNAs and circRNAs. However, we only identified 15 DEMs through sequencing, possibly because of batch effect. In order to screen more DEMs, we performed batch-correction methods to eliminate the effect as much as possible. Consequently, we only screened significantly upregulated miRNAs. As Brophy et al. (Brophy et al., 2018) also predicted relatively low DEMs in the menisci dissected from TKA patients compared with those in arthroscopic partial meniscectomy (APM)-derived menisci, it is possible that only a few DEMs can be detected in degenerative menisci. Interestingly, miR-146-5p was specifically upregulated in OA006_IL-1β (46-fold changes). The differences between the sequences might contribute to meniscus sample heterogeneity between patients as we discussed before, and the inflammatory cytokine treatment might act diversely between different primary meniscus cells. However, after qRT-PCR validation, miR-146-5p was upregulated in all other three samples, suggesting that miR-146-5p is actually upregulated upon IL-1β stimulation. Therefore, we believe that a meniscus database for OA patients needs to be constructed in the future in order to cut down errors brought by sample heterogeneity.

LncRNAs over 200 nucleotides in length are also known to be derived from mammalian genomes and have been studied as a decoy for miRNA to combine with and inhibit expression (Ponting et al., 2009; Wang and Chang, 2011). For instance, Wang et al. (2019) demonstrated that lncRNA FOXD2-AS1 increased the expression levels of *TLR4* by sponging with miR-27a-3p, thereby inducing chondrocyte proliferation. On the other hand, knockdown of lncRNA-like lncRNA MF12-AS1 leads to miR-130a-3p upregulation and therefore interferes with the expression of *TCF4*, which results in increased chondrocyte viability and inhibition of apoptosis, inflammatory response, and extracellular matrix (ECM) degeneration in OA (Luo et al., 2020). All these studies suggest that the sponging function of lncRNA is an important mechanism within OA cartilage. In our present work, we screened out 56 DELs in IL-1β-treated degenerative menisci versus non-IL-1β-treated degenerative menisci. A previous study identified 10 DEL results using TKA to obtain degenerative menisci versus APM to garner a traumatic meniscus (Brophy et al., 2018). LncRNA expression differences might possibly be based on the divergence of OA patients or the conspicuous inflammatory effect of IL-1β. Based on our DEL results, we performed lncRNA–miRNA–mRNA network prediction by applying the RNAhybrid algorithm, and lncRNA LOC107986251 possessed the greatest amount of ceRNA networks in degenerative menisci with IL-1β treatment. Furthermore, we overlapped miRanda and RNAhybrid results to screen out the most specific lncRNA regulatory network. Six lncRNA–miRNA–mRNA ceRNA networks are potentially regulated in the pathogenesis of meniscus OA. Among these, *SESN3*, which was previously investigated for supporting chondrocyte homeostasis and is suppressed in OA cartilage (Shen et al., 2017), was also downregulated by the modulation of the LOC107986251-hsa-miR-212-5p-SESN3 network in OA-induced degenerative menisci. The qRT-PCR validation supported this result. Therefore, the downregulation of lncRNA LOC107986251 might induce miR-212-5p expression and inhibit SESN3 expression, leading to the meniscus and cartilage degenerative process, suggesting a potential crosslink between menisci and cartilage during OA. Nonetheless, deeper mechanistic validation is needed to confirm this hypothesis.

CircRNAs are novel regulatory RNAs that have been recently investigated in OA chondrocytes. Recently, CircSERPINE2-miR-1271-5P-E26 transformation-specific-related gene axis was found to have a strong protective effect against OA by inhibiting chondrocyte apoptosis and ECM degeneration (Shen et al., 2019). This finding suggests that the miRNA-sponging function of circRNA is a potential source of OA development. However, few studies on circRNA have been employed for OA-induced degenerative menisci. Hence, as whole-transcriptome sequencing can simultaneously detect circRNA expression as well, we identified novel DECs in degenerative menisci with or without IL-1β stimulation, including 56 significantly upregulated and 34 significantly downregulated circRNAs. Similarly, we predicted the circRNA–miRNA–mRNA network using the same method as the prediction of the lncRNA ceRNA network, discovering that hsa_circ_0018069 possessed the highest number of networks. Specifically, only one ceRNA regulation network was identified by overlapping the miRanda and RNAhybrid outputs. The downregulation of hsa_circ_0018069 retarded its sponging effect with hsa-miR-147b-3p so that the expression of TJP2 was dysregulated during the OA process in the menisci. Conversely, the hsa_circ_0018069-miR-147b-3p-TJP2 axis might also serve as protection against meniscus degeneration in OA, like circSERPINE2 on the cartilage. It has been reported that hsa_circ_0018069 expression is inhibited in bladder cancer tissues and may serve as a clinical biomarker for early bladder cancer. *TJP2* has been studied previously. However, none of these network components have been further evaluated in menisci or cartilage during OA, which might suggest a possible novel regulatory pathway in meniscus degeneration. Our qRT-PCR validation confirmed the predicted expression pattern of hsa_circ_0018069-miR-147B-3p-TJP2 in the menisci with IL-1β stimulation, yet this axis still requires further verification *in vitro* and *in vivo*.

Menisci have been largely reported to have an important role in OA progress, and destabilization of the medial meniscus (DMM) model is a common OA model for mice (Berthiaume et al., 2005; Hunter et al., 2006; Murphy et al., 2019). However, whether meniscus degeneration or meniscus-specific biomarkers forecast the onset of OA or the severity of OA remains unknown. Hence, aside from ncRNAs, we screened mRNAs by overlapping the three constructed meniscus databases. *LCN2* and *RAB27B* exhibited significant upregulation during OA in menisci. *LCN2*, also known as neutrophil gelatinase-associated lipocalin, has recently been identified as a pro-inflammatory adipokine in OA chondrocytes. However, *LCN2* overexpression via adenovirus injection into the murine joint did not trigger OA pathology, and global *LCN2* knockout mice showed no restoration of cartilage in DMM-induced mice (Choi and Chun, 2017). This might imply that early-stage OA was triggered via *LCN2* activation in the menisci but not in the chondrocytes. On the other hand, no studies have been conducted concerning *RAB27B* in OA; however, a recent investigation revealed that *RAB27B* acts as a downstream mediator of *HIF-2α* to regulate the formation of the vascular network (Bhurke et al., 2020). Intriguingly, *RAB27B* had also been predicted to be highly expressed in DegP in a previous study (Sun et al., 2020). Since meniscus degeneration after trauma or tear is of high relevance to the avascular traits in the white zone of the menisci during OA, it is reasonable to believe that *RAB27B* might contribute to increasing avascular traits during meniscus degeneration in OA. Following immunohistochemical studies on menisci derived from human suggested that these two meniscus-specific biomarkers correlated with OA severity. *In vivo* study showed meniscus-specific *LCN2* and *RAB27B* remarkably upregulated at the age of 26 weeks (6 months) in mice and specifically distributed in the internal zone of menisci. Strangely, mice at the age of 52 weeks (1 year) did not show highly positive *LCN2* and *RAB27B* in menisci. We hypothesize that *LCN2* and *RAB27B* might act as a warning and protective signal for OA in murine knee. As the aging and OA develops, their expression begins to fade away. In OA patients, on the other hand, meniscus-specific *LCN2* and *RAB27B* remain consistently expressed and play the part of OA severity prediction. Since menisci or cartilage from early-stage OA patients were usually not able to be obtained, the relation between *LCN2* and *RAB27B* and the period of OA prediction in human remain blurry and require further analysis. Anyway, both of these results are promising for the study of the mechanism underlying meniscus degeneration during OA.

The main strength of this study is to use the advanced high throughout sequence methods—whole-transcriptome sequence to predict the potential mRNA and noncoding RNA, which is more comprehensive than mere RNA sequence. Furthermore, based on the whole-transcriptome sequence data, we overlapped miRanda and RNAhybrid predicting algorithm, and we were able to predict two specific RNA regulatory axis—lncRNA LOC107986251-miR-212-5p-SESN3 and hsa_circ_0018069-miR-147b-3p-TJP2—which could be a novel target for the early treatment of degenerative menisci. More importantly, by combining different databases, we were also able to discover two highly specific markers, LCN2 and RAB27B, which are also highly specific since these two biomarkers were both significantly altered in three different databases of degenerative meniscus.

Although several novel findings were proposed in the OA-induced degenerative meniscus, this study has several limitations. First of all, IL-1β diluent was not used as an exact positive control, while we applied refreshed medium instead. Moreover, following PCA, we have discovered that sample OA006_NC exhibited heterogeneity compared with OA004_NC and OA008_NC (Supplemental Figure S1). This phenomenon might contribute to slight influence on the following sequence results, and we will discuss it in our limitations. Hence, a larger database of degenerative menisci from OA patients and even normal menisci should be built in order to offer a global understanding of distinct genes and ncRNA expression during meniscus degeneration, so that further investigation of meaningful clinical biomarkers for OA patients can be efficiently performed. It could also cut down some examination errors brought by sample heterogeneity as we mentioned above. Another limitation is the highly rigorous selection for lncRNA and circRNA target prediction by overlapping miRanda, RNAhybrid algorithm, and miRNA sequencing, which might contribute to relatively less ceRNA network results. Nevertheless, it also helps us to identify highly specific ceRNA regulatory pathways during meniscus degeneration during OA. In addition, we performed simple validation on the differential expression of each ncRNA and mRNA using qRT-PCR. To further confirm their specific mechanism and function in the degenerative process of OA menisci, more in-depth research into significantly upregulated and downregulated ncRNAs should be performed.

In summary, this study illustrated a transcriptome profile of OA menisci by a whole-transcriptome sequencing method and specifically identified two highly specific ceRNA networks regulated by lncRNA LOC107986251 and hsa_circ_0018069, which possibly play an important role during the meniscal degeneration process, and two potential mRNA biomarkers, *LCN2* and *RAB27B*, in the meniscus for future OA diagnosis. All these bioinformatics results could be of value to researchers seeking to understand the underlying mechanism of meniscus degeneration in OA, hence exploiting new diagnostic biomarkers for early-stage OA clinical lab tests and potential therapeutic targets for treating OA. Furthermore, the relationship between degenerative knee menisci and cartilage during the OA process could also be explored based on the present study. However, additional efforts are merited for DEGs, DEMs, DELs, DECs, and ceRNA networks to achieve the aforementioned goals.

## Data Availability

The data presented in the study are deposited in the GEO repository, accession number GSE185064, accession number GSE171652.
