# Supplementary material for: Whole-Transcriptome Sequence of Degenerative Meniscus Cells Unveiling Diagnostic Markers and Therapeutic Targets for Osteoarthritis
Source: Front Genet. 2021 Oct 15;12:754421. doi: 10.3389/fgene.2021.754421 (PMC8554121; doi:10.3389/fgene.2021.754421)
Supplement: Supplementary file 1 [file DataSheet4.DOCX]

**Supplemental Table 4. Eighty-nine differentially expressed circular RNAs (circRNAs; OA menisci with versus without IL-1β treatment.**$\mathbf{log}_{\boldsymbol{2}} \mathbf{FC}$ **< 1, FDR < 0.05)**

| **CircbaseID** | **GeneName** | **log2FC** | **FoldChange** | **FDR** | **Style** |
| --- | --- | --- | --- | --- | --- |
|  | MMP1 | 12.90981466 | 7695.581672 | 0 | up |
| hsa_circ_0094044\|chr10:70719561-70721913+\|ENST00000354185.4\|DDX21 | DDX21 | 5.926404705 | 60.81708253 | 5.28806E-07 | up |
| hsa_circ_0002686\|chr4:40936472-40947090-\|NM_004307\|APBB2 | APBB2 | 4.729775141 | 26.53408953 | 0.038945836 | up |
| hsa_circ_0007221\|chr10:75331178-75336119-\|NM_152586\|USP54 | USP54 | 4.387929873 | 20.93623144 | 0.009647879 | up |
|  | OGDH | 4.325700071 | 20.05235911 | 0.039682222 | up |
| hsa_circ_0005221\|chr17:79563141-79575848-\|NM_017921\|NPLOC4 | NPLOC4 | 4.282670417 | 19.46311089 | 0.002243661 | up |
| hsa_circ_0005890\|chr9:4191785-4286523-\|NM_001042413\|GLIS3 | GLIS3 | 4.256239189 | 19.109779 | 7.84125E-06 | up |
|  | HERC4 | 4.058070111 | 16.65715503 | 0.000717641 | up |
| hsa_circ_0005937\|chr7:111575594-111585853-\|NM_014705\|DOCK4 | DOCK4 | 4.005631692 | 16.06257952 | 0.011932401 | up |
| hsa_circ_0006768\|chr6:87925620-87955362+\|NM_015021\|ZNF292 | ZNF292 | 3.993458944 | 15.92762157 | 0.046815353 | up |
| hsa_circ_0008917\|chr9:125719289-125752492+\|NM_012197\|RABGAP1 | RABGAP1 | 3.909709887 | 15.02934142 | 0.032024403 | up |
| hsa_circ_0007208\|chr3:73004248-73016870+\|NM_001080393\|GXYLT2 | GXYLT2 | 3.865536891 | 14.57614075 | 0.00058542 | up |
| hsa_circ_0003251\|chr12:1003727-1006847+\|NM_001184985\|WNK1 | WNK1 | 3.6432936 | 12.49512648 | 0.0008458 | up |
| hsa_circ_0007037\|chr9:88918000-88919862-\|NM_024617\|ZCCHC6 | ZCCHC6 | 3.6432936 | 12.49512648 | 0.000848505 | up |
| hsa_circ_0002482\|chr9:103082546-103084053-\|NM_017746\|TEX10 | TEX10 | 3.608553641 | 12.19783872 | 0.034501903 | up |
| hsa_circ_0008602\|chr11:106849344-106856857-\|NM_000855\|GUCY1A2 | GUCY1A2 | 3.536285696 | 11.60187195 | 0.043831223 | up |
| hsa_circ_0008581\|chr13:52971366-52976816-\|NM_018676\|THSD1 | THSD1 | 3.469549255 | 11.07741426 | 0.021494969 | up |
|  | MTHFD2L | 3.446453037 | 10.90148704 | 0.001656272 | up |
| hsa_circ_0012397\|chr1:51001040-51061888-\|NM_007051\|FAF1 | FAF1 | 3.425023471 | 10.74075476 | 0.006386661 | up |
| hsa_circ_0002843\|chr1:217783662-217787544-\|NM_018040\|GPATCH2 | GPATCH2 | 3.211372078 | 9.26231022 | 0.011193487 | up |
|  | ACAP2 | 2.991793078 | 7.954620357 | 0.000347047 | up |
| hsa_circ_0045202\|chr17:61655830-61657304+\|NM_005828\|DCAF7 | DCAF7 | 2.988279822 | 7.935272803 | 0.000956071 | up |
| hsa_circ_0006380\|chr15:57523349-57526305+\|NM_207037\|TCF12 | TCF12 | 2.987125903 | 7.928928429 | 0.009379615 | up |
| hsa_circ_0088829\|chr9:131329016-131331176+\|NM_001130438\|SPTAN1 | SPTAN1 | 2.971549266 | 7.843781056 | 0.000360707 | up |
| hsa_circ_0133991\|chr7:24663281-24681487+\|ENST00000396475.2\|MPP6 | MPP6 | 2.93724939 | 7.659495603 | 0.013131012 | up |
| hsa_circ_0001487\|chr5:58284319-58289292-\|NM_001104631\|PDE4D | PDE4D | 2.842819745 | 7.174208853 | 3.04373E-06 | up |
|  | na | 2.827247271 | 7.097186759 | 0.006198659 | up |
| hsa_circ_0007606\|chr4:146791396-146813567-\|NM_178835\|ZNF827 | ZNF827 | 2.752088118 | 6.736915096 | 0.002501859 | up |
| hsa_circ_0075157\|chr5:176618884-176631293+\|NM_022455\|NSD1 | NSD1 | 2.749296039 | 6.72388961 | 0.047531686 | up |
| hsa_circ_0069227\|chr4:15626874-15646331-\|NR_036464\|FBXL5 | FBXL5 | 2.402439206 | 5.286962913 | 0.02554076 | up |
| hsa_circ_0002622\|chr3:169854206-169896726-\|NM_024947\|PHC3 | PHC3 | 2.259567431 | 4.788478853 | 0.018203578 | up |
| hsa_circ_0006750\|chr10:96234424-96260084+\|NM_015188\|TBC1D12 | TBC1D12 | 2.22841995 | 4.686204621 | 0.000878892 | up |
| hsa_circ_0007933\|chrX:10534927-10535643-\|NM_000381\|MID1 | MID1 | 2.225680919 | 4.677316059 | 0.03990265 | up |
| hsa_circ_0076560\|chr6:43514307-43516197-\|NM_020750\|XPO5 | XPO5 | 2.207623516 | 4.619137575 | 0.020388325 | up |
| hsa_circ_0002198\|chr6:136472297-136476896+\|NM_018945\|PDE7B | PDE7B | 2.138745425 | 4.40378924 | 0.002287208 | up |
| hsa_circ_0004662\|chr6:160103505-160109274-\|NM_001024465\|SOD2 | SOD2 | 2.091670248 | 4.262412588 | 1.1018E-07 | up |
| hsa_circ_0001358\|chr3:169694733-169706147+\|NM_003262\|SEC62 | SEC62 | 2.079428136 | 4.226396547 | 5.85745E-10 | up |
| hsa_circ_0005505\|chr12:66597490-66622150+\|NM_007199\|IRAK3 | IRAK3 | 2.069053227 | 4.19611212 | 0.00493004 | up |
| hsa_circ_0003315\|chr16:74493579-74497377-\|NR_027264\|GLG1 | GLG1 | 2.068646987 | 4.194930727 | 0.038399111 | up |
|  | MEDAG | 2.035381209 | 4.099310327 | 0.002126552 | up |
| hsa_circ_0005309\|chr14:105718843-105739231-\|NM_001519\|BRF1 | BRF1 | 2.009913444 | 4.027580554 | 0.008554898 | up |
| hsa_circ_0111334\|chr1:179087721-179091002-\|NM_007314\|ABL2 | ABL2 | 1.993430897 | 3.981827983 | 2.26986E-05 | up |
| hsa_circ_0001833\|chr8:145245686-145255444+\|NM_001099281\|HEATR7A | MROH1 | 1.988495642 | 3.968229985 | 0.044371757 | up |
| hsa_circ_0000660\|chr15:94899365-94945248+\|NM_001159643\|MCTP2 | MCTP2 | 1.948316499 | 3.859239287 | 0.000213516 | up |
|  | PHF20 | 1.943794974 | 3.847163046 | 0.00586886 | up |
|  | TTC7A | 1.862764154 | 3.637038391 | 2.79036E-06 | up |
| hsa_circ_0003232\|chr16:53907697-53913899+\|NM_001080432\|FTO | FTO | 1.846270374 | 3.595694308 | 0.024452132 | up |
| hsa_circ_0002166\|chr1:22074630-22078108-\|NM_032236\|USP48 | USP48 | 1.745277884 | 3.352594234 | 2.9365E-06 | up |
| hsa_circ_0001971\|chr7:23015828-23023664-\|NM_032581\|FAM126A | FAM126A | 1.705705021 | 3.261882967 | 0.004376446 | up |
| hsa_circ_0003893\|chr15:81229014-81230320+\|NM_018689\|KIAA1199 | CEMIP | 1.572942148 | 2.975108221 | 0.007081542 | up |
| hsa_circ_0003692\|chr3:171969049-172028671+\|NM_022763\|FNDC3B | FNDC3B | 1.556685664 | 2.941772465 | 0.028517837 | up |
| hsa_circ_0000053\|chr1:36826821-36828257-\|NM_032017\|STK40 | STK40 | 1.551770327 | 2.931766747 | 9.44528E-05 | up |
| hsa_circ_0008618\|chr4:151719232-151738409-\|NM_001199282\|LRBA | LRBA | 1.487654872 | 2.804327558 | 0.041442137 | up |
| hsa_circ_0024085\|chr11:95825055-95826681-\|NM_032427\|MAML2 | MAML2 | 1.405602224 | 2.64928348 | 7.98632E-06 | up |
| hsa_circ_0005942\|chr18:42281139-42283072+\|NM_015559\|SETBP1 | SETBP1 | 1.137068586 | 2.199336854 | 0.042469461 | up |
| hsa_circ_0000467\|chr13:21742126-21742538-\|NM_145061\|SKA3 | SKA3 | -1.141073754 | 0.453421984 | 0.001159951 | down |
|  | BNC2 | -1.298780062 | 0.406469762 | 0.025902261 | down |
| hsa_circ_0020078\|chr10:116590610-116596005+\|NM_020940\|FAM160B1 | FAM160B1 | -1.333527999 | 0.396796719 | 0.036409876 | down |
| hsa_circ_0098286\|chr12:28408513-28460682+\|ENST00000545336.1\|CCDC91 | CCDC91 | -1.373730403 | 0.385892149 | 0.017336057 | down |
| hsa_circ_0008583\|chr3:196817782-196846401-\|NM_004087\|DLG1 | DLG1 | -1.434346227 | 0.370014516 | 0.007607512 | down |
| hsa_circ_0002970\|chr15:81199003-81201647+\|NM_018689\|KIAA1199 | CEMIP | -1.806310988 | 0.285921103 | 0.01867079 | down |
|  | HIBADH | -1.962011306 | 0.256670375 | 0.031679502 | down |
| hsa_circ_0005315\|chr2:8910799-8917022-\|NM_020738\|KIDINS220 | KIDINS220 | -1.964302581 | 0.256263057 | 0.004778604 | down |
| hsa_circ_0008037\|chr19:34922765-34925873+\|NM_005499\|UBA2 | UBA2 | -2.005118164 | 0.249114661 | 0.036780474 | down |
|  | WDR20 | -2.010653802 | 0.248160637 | 0.01779842 | down |
| hsa_circ_0030377\|chr13:61013821-61060082+\|NM_001146070\|TDRD3 | TDRD3 | -2.096337899 | 0.233851097 | 0.026545774 | down |
| hsa_circ_0001466\|chr5:6604250-6605521-\|NM_001193455\|NSUN2 | NSUN2 | -2.23563124 | 0.212328327 | 0.047415129 | down |
|  | NAA35 | -2.23563124 | 0.212328327 | 0.023751186 | down |
|  | UHRF2 | -2.298840026 | 0.203226434 | 0.003755572 | down |
| hsa_circ_0025693\|chr12:27075548-27081838-\|NM_018164\|C12orf11 | ASUN | -2.577676035 | 0.167510561 | 0.048898912 | down |
| hsa_circ_0007090\|chr7:90001468-90007525+\|NM_033107\|GTPBP10 | GTPBP10 | -2.609398132 | 0.163867524 | 0.017672714 | down |
|  | PRKCA | -2.900571407 | 0.133918631 | 0.00616812 | down |
| hsa_circ_0008244\|chr4:122725776-122731254+\|NM_001034194\|EXOSC9 | EXOSC9 | -2.93099535 | 0.131124089 | 0.025643836 | down |
| hsa_circ_0046965\|chr18:12356692-12371690-\|NM_006796\|AFG3L2 | AFG3L2 | -2.953004029 | 0.129138938 | 0.003378546 | down |
| hsa_circ_0003183\|chr8:61496766-61504528+\|NM_002865\|RAB2A | RAB2A | -2.955117998 | 0.12894985 | 2.19222E-06 | down |
| hsa_circ_0018069\|chr10:30315031-30318795-\|NM_020848\|KIAA1462 | KIAA1462 | -3.030238363 | 0.122407311 | 0.013549711 | down |
| hsa_circ_0095749\|chr11:34097782-34098189+\|NM_005898\|CAPRIN1 | CAPRIN1 | -3.119609042 | 0.115054631 | 0.01802915 | down |
| hsa_circ_0100857\|chr13:75930282-75936743-\|ENST00000377636.3\|TBC1D4 | TBC1D4 | -3.153527247 | 0.112381211 | 0.01915973 | down |
| hsa_circ_0006758\|chr1:165859440-165860559+\|NM_012474\|UCK2 | UCK2 | -3.346738739 | 0.09829496 | 2.59409E-05 | down |
|  | GK5 | -3.568980577 | 0.084261618 | 0.012111185 | down |
|  | EML1 | -3.692366357 | 0.077354747 | 6.41476E-06 | down |
| hsa_circ_0081892\|chr7:106921744-106938791-\|NM_001161520\|COG5 | COG5 | -3.856073569 | 0.069056759 | 0.037101636 | down |
| hsa_circ_0017310\|chr1:246754813-246755243+\|NM_152609\|CNST | CNST | -3.896694672 | 0.067139487 | 0.000287359 | down |
| hsa_circ_0008541\|chrX:149631037-149642074+\|NM_005491\|MAMLD1 | MAMLD1 | -3.983652785 | 0.063212217 | 0.03986008 | down |
| hsa_circ_0006482\|chr16:67070541-67116211+\|NM_022845\|CBFB | CBFB | -4.179910499 | 0.05517236 | 0.026327105 | down |
| hsa_circ_0088095\|chr9:115200710-115204050+\|NM_032303\|HSDL2 | HSDL2 | -4.208216387 | 0.05410042 | 0.022640062 | down |
| hsa_circ_0010444\|chr1:21083658-21106404-\|NM_016287\|HP1BP3 | HP1BP3 | -4.239899833 | 0.052925257 | 0.014589315 | down |
| hsa_circ_0101384\|chr14:104168410-104170593+\|ENST00000555832.1\|XRCC3 | na | -4.527628799 | 0.043355872 | 0.000246027 | down |
| hsa_circ_0000277\|chr11:14793482-14810788+\|NM_000922\|PDE3B | PDE3B | -4.716016196 | 0.038048511 | 9.70577E-05 | down |
