# Supplementary figures and images for: Whole-Transcriptome Sequence of Degenerative Meniscus Cells Unveiling Diagnostic Markers and Therapeutic Targets for Osteoarthritis
Source: Front Genet. 2021 Oct 15;12:754421. doi: 10.3389/fgene.2021.754421 (PMC8554121; doi:10.3389/fgene.2021.754421)

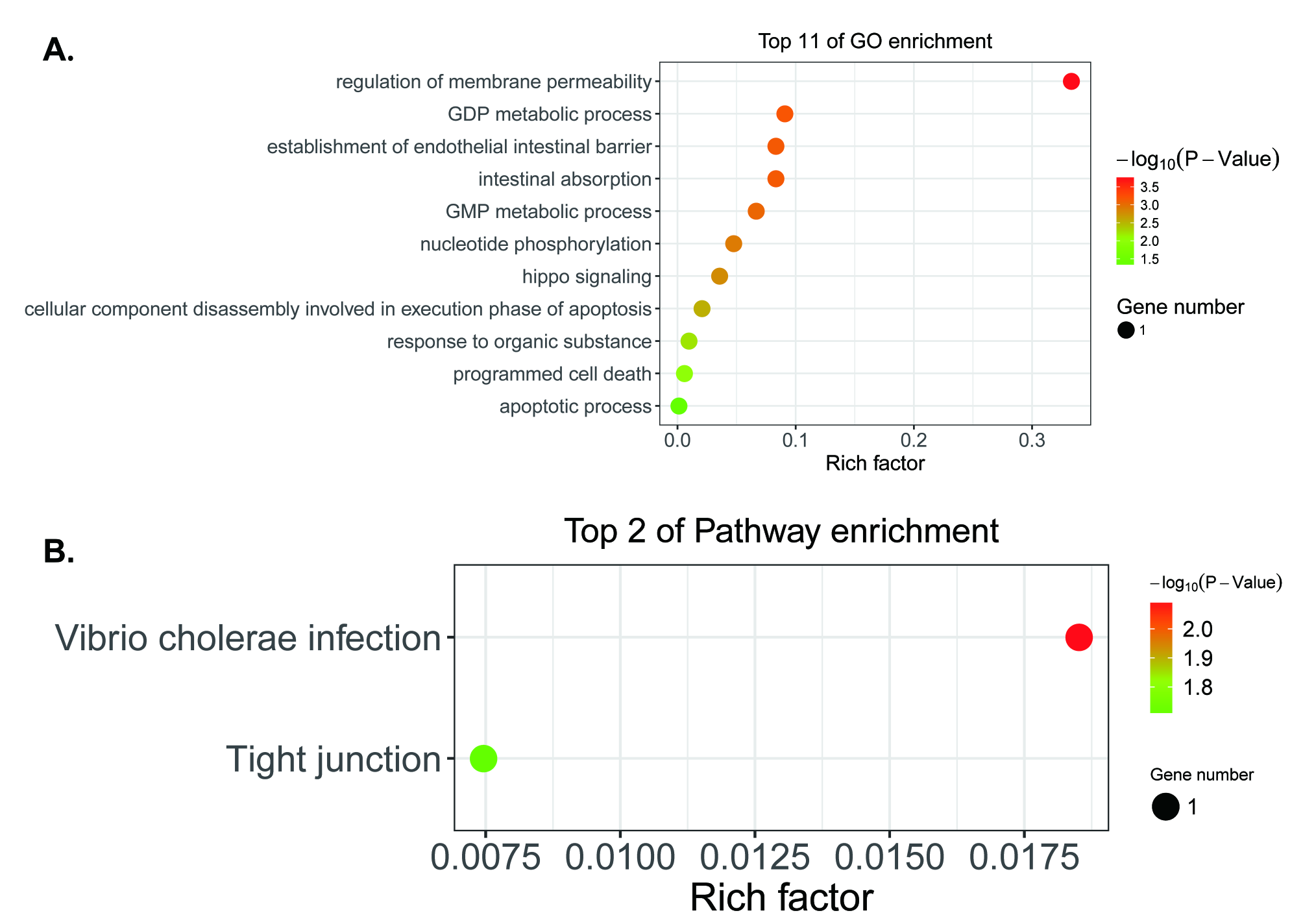

Supplement: Supplementary file 2 [file Image3.TIF]

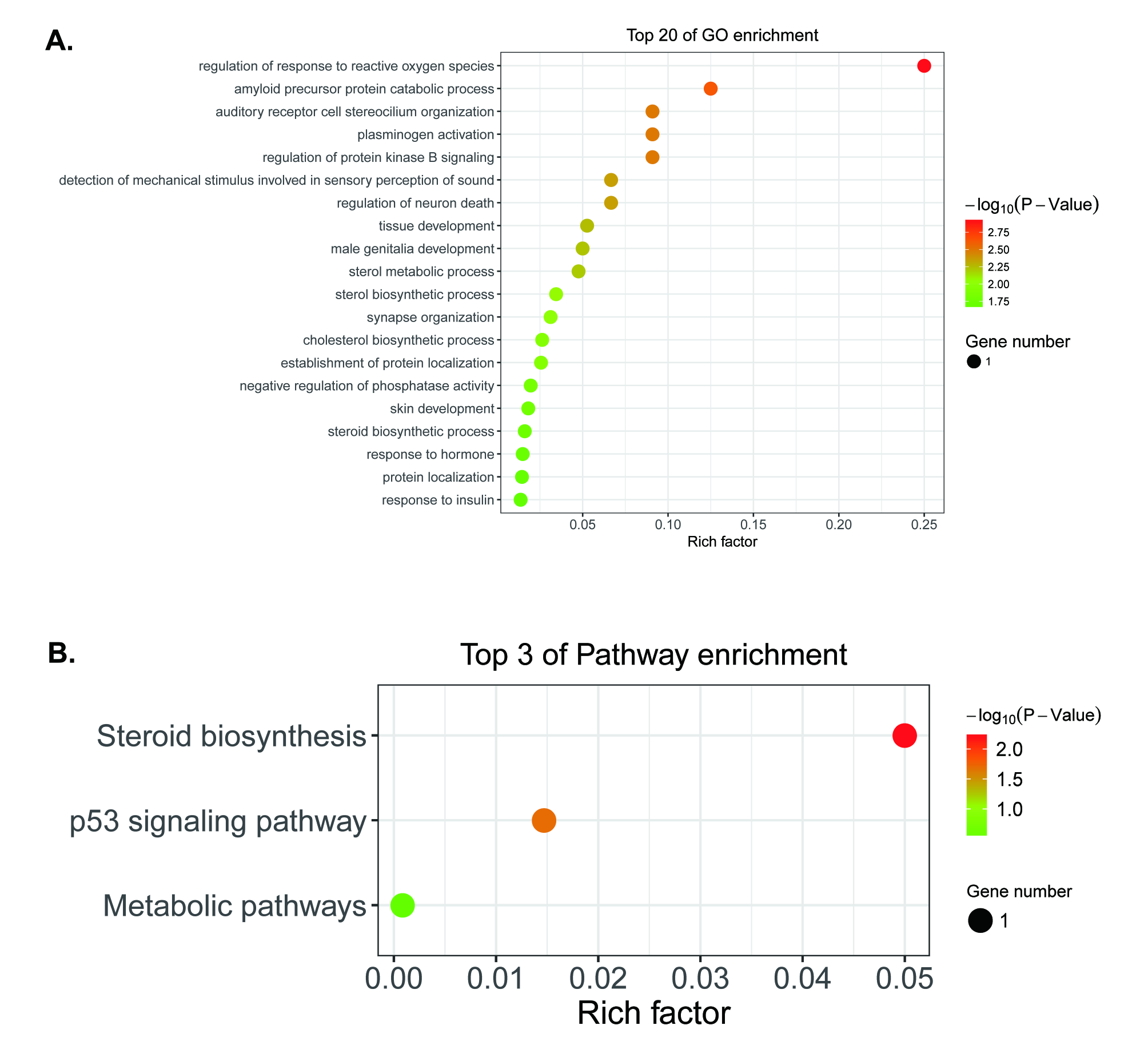

Supplement: Supplementary file 4 [file Image2.TIF]

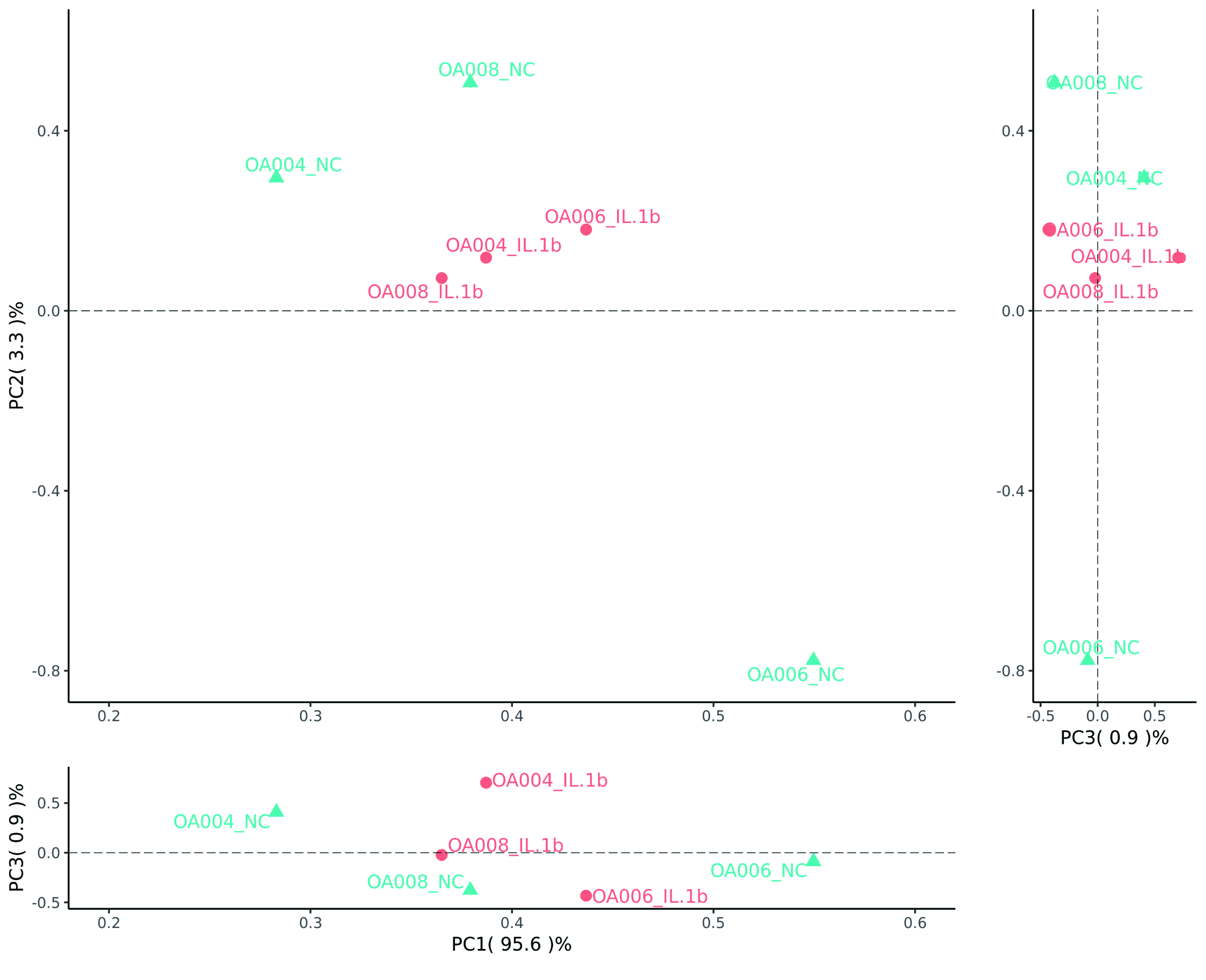

Supplement: Supplementary file 5 [file Image1.TIF]
