# Supplementary material for: Whole-Transcriptome Sequence of Degenerative Meniscus Cells Unveiling Diagnostic Markers and Therapeutic Targets for Osteoarthritis
Source: Front Genet. 2021 Oct 15;12:754421. doi: 10.3389/fgene.2021.754421 (PMC8554121; doi:10.3389/fgene.2021.754421)
Supplement: Supplementary file 3 [file DataSheet3.DOCX]

**Supplemental Table 3. Fifty-six differentially expressed long non-coding RNAs. (lncRNAs; OA menisci with versus without IL-1β treatment.**$\mathbf{log}_{\boldsymbol{2}} \mathbf{FC}$ **< 1, FDR < 0.05)**

| **AccID** | **log2FC** | **FoldChange** | **FDR** | **IsoLength** | **type_of_gene** | **KeggID** | **Description** |
| --- | --- | --- | --- | --- | --- | --- | --- |
| BRE-AS1 | 1.018577299 | 2.025920131 | 0.047374879 | 1659 | ncRNA | hsa:100302650 | BRE antisense RNA 1 |
| DARS-AS1 | 1.278737564 | 2.426265726 | 2.05255E-05 | 881 | ncRNA | hsa:101928243 | DARS antisense RNA 1 |
| DLGAP1-AS1 | 1.18997354 | 2.281485587 | 3.55661E-05 | 993 | ncRNA | hsa:649446 | DLGAP1 antisense RNA 1 |
| DNM1P9 | -5.002077354 | 0.031205035 | 0.013381184 | 1265 | pseudo |  | DNM1 pseudogene 9 |
| FTH1P10 | 1.249081226 | 2.37690003 | 0 | 919 | pseudo |  | ferritin, heavy polypeptide 1 pseudogene 10 |
| FTH1P15 | 1.776107545 | 3.425008445 | 0.048723759 | 456 | pseudo |  | ferritin, heavy polypeptide 1 pseudogene 15 |
| FTH1P20 | 2.302806391 | 4.934166471 | 1.45632E-05 | 861 | pseudo |  | ferritin, heavy polypeptide 1 pseudogene 20 |
| FTH1P23 | 1.365579488 | 2.576798075 | 0.019076698 | 865 | pseudo |  | ferritin, heavy polypeptide 1 pseudogene 23 |
| FTH1P7 | 1.280321402 | 2.428930822 | 1.25174E-07 | 930 | pseudo |  | ferritin, heavy polypeptide 1 pseudogene 7 |
| FTH1P8 | 1.338359936 | 2.528636984 | 0.015724767 | 912 | pseudo |  | ferritin, heavy polypeptide 1 pseudogene 8 |
| GBP1P1 | 3.180686354 | 9.067383814 | 0.000533596 | 1234 | pseudo | hsa:400759 | guanylate binding protein 1, interferon-inducible pseudogene 1 |
| HMGN2P46 | 2.569768092 | 5.937139831 | 0.000409656 | 1927 | pseudo | hsa:283651 | high mobility group nucleosomal binding domain 2 pseudogene 46 |
| KRT87P | 2.284872112 | 4.873209051 | 0.011274326 | 1257 | pseudo |  | keratin 87 pseudogene |
| LINC00473 | 4.026683277 | 16.29868061 | 0.014921886 | 1822 | ncRNA | hsa:90632 | long intergenic non-protein coding RNA 473 |
| LINC01588 | 1.956538037 | 3.881294857 | 0.001113767 | 4835 | ncRNA | hsa:283551 | chromosome 14 open reading frame 182 |
| LOC100130417 | -1.246829781 | 0.421373128 | 1.70397E-12 | 1506 | ncRNA | hsa:100130417 | uncharacterized LOC100130417 |
| LOC100505622 | 3.630979206 | 12.38892588 | 0 | 685 | ncRNA | hsa:100505622 | uncharacterized LOC100505622 |
| LOC100506178 | 4.149288847 | 17.7443626 | 2.90892E-05 | 2434 | ncRNA | hsa:100506178 | uncharacterized LOC100506178 |
| LOC100506188 | 1.984423422 | 3.957044864 | 0.000285071 | 1460 | ncRNA | hsa:100506188 | uncharacterized LOC100506188 |
| LOC100507639 | 1.901911094 | 3.737079083 | 0.004788178 | 1025 | lncRNA | hsa:100507639 | uncharacterized LOC100507639 |
| LOC101232810 | -1.582527888 | 0.333896323 | 0.021895449 | 1895 | pseudo |  | stathmin-like 3 pseudogene |
| LOC101929475 | 1.099278733 | 2.142475539 | 0.000904023 | 3526 | ncRNA | hsa:101929475 | uncharacterized LOC101929475 |
| LOC102723741 | 2.659078601 | 6.316295208 | 0.033827824 | 2153 | ncRNA | hsa:102723741 | uncharacterized LOC102723741 |
| LOC102724015 | -1.296344329 | 0.407156593 | 0.005059908 | 2114 | ncRNA | hsa:102724015 | uncharacterized LOC102724015 |
| LOC105371401 | 1.338100728 | 2.528182707 | 0.009442282 | 4995 | ncRNA |  | uncharacterized LOC105371401 |
| LOC105371453 | 1.219578494 | 2.328786681 | 0.022681256 | 9534 | ncRNA |  | uncharacterized LOC105371453 |
| LOC105372639 | 1.852817008 | 3.612047858 | 0.001616858 | 3550 | ncRNA |  | uncharacterized LOC105372639 |
| LOC105372663 | 3.508009098 | 11.37669103 | 0.001957005 | 1000 | ncRNA |  | uncharacterized LOC105372663 |
| LOC105375153 | 2.193258409 | 4.573372431 | 0.006818579 | 1227 | ncRNA |  | uncharacterized LOC105375153 |
| LOC105375511 | 1.737385048 | 3.334302618 | 0.040838651 | 1299 | ncRNA |  | uncharacterized LOC105375511 |
| LOC105376144 | 3.412536623 | 10.64819228 | 0.029049598 | 958 | ncRNA |  | uncharacterized LOC105376144 |
| LOC105377272 | 5.232401051 | 37.59323208 | 0.047328411 | 4148 | ncRNA |  | uncharacterized LOC105377272 |
| LOC105377275 | 3.94968207 | 15.45157579 | 2.33147E-14 | 3231 | ncRNA |  | uncharacterized LOC105377275 |
| LOC105378085 | 2.355277153 | 5.116925234 | 0.036293144 | 3894 | ncRNA |  | uncharacterized LOC105378085 |
| LOC105378114 | 4.467299825 | 22.12031194 | 0.035082501 | 8906 | ncRNA |  | uncharacterized LOC105378114 |
| LOC105378917 | 2.532641384 | 5.786301043 | 1.73195E-14 | 5383 | ncRNA |  | uncharacterized LOC105378917 |
| LOC105379771 | 5.482497537 | 44.70912905 | 8.68857E-05 | 1203 | ncRNA |  | uncharacterized LOC105379771 |
| LOC107984523 | 2.427731784 | 5.380468442 | 1.70008E-12 | 3978 | lncRNA |  |  |
| LOC107985321 | -2.577056082 | 0.167582559 | 0.018602364 | 640 | ncRNA |  |  |
| LOC107986153 | 1.517738728 | 2.863418871 | 0.002269352 | 3146 | ncRNA |  |  |
| LOC107986251 | -1.767258035 | 0.293766535 | 1.30351E-12 | 6972 | ncRNA |  |  |
| LOC107986637 | -1.82643396 | 0.281960708 | 0.006403461 | 1715 | ncRNA |  |  |
| LOC107986874 | 2.170249407 | 4.501011989 | 0.002494362 | 6022 | ncRNA |  |  |
| LOC200772 | -1.90602489 | 0.266826731 | 0.015374618 | 2274 | ncRNA | hsa:200772 | uncharacterized LOC200772 |
| LOC541472 | 4.699484761 | 25.98279562 | 1.69031E-06 | 1364 | ncRNA |  | uncharacterized LOC541472 |
| LUCAT1 | 2.743034586 | 6.694770433 | 2.25136E-09 | 890 | ncRNA | hsa:100505994 | lung cancer associated transcript 1 (non-protein coding) |
| MSC-AS1 | 1.025384324 | 2.035501555 | 8.55155E-08 | 4802 | ncRNA | hsa:100132891 | uncharacterized LOC100132891 |
| MT1L | 1.421152547 | 2.677993664 | 8.83071E-13 | 426 | pseudo | hsa:4500 | metallothionein 1L (gene/pseudogene) |
| MT2P1 | 4.692332914 | 25.8543103 | 6.48977E-07 | 416 | pseudo |  | metallothionein 2 pseudogene 1 |
| NAMPTP1 | 2.085740924 | 4.244930472 | 0.00024943 | 2501 | pseudo |  | nicotinamide phosphoribosyltransferase pseudogene 1 |
| PACERR | 1.606294438 | 3.044688086 | 0.017036926 | 793 | ncRNA |  | PTGS2 antisense NFKB1 complex-mediated expression regulator RNA |
| RBPMS-AS1 | 1.17815623 | 2.262873959 | 4.64353E-10 | 909 | ncRNA | hsa:100128750 | RBPMS antisense RNA 1 |
| RN7SL124P | 4.594798862 | 24.16419221 | 2.37865E-05 | 273 | pseudo |  | RNA, 7SL, cytoplasmic 124, pseudogene |
| RN7SL220P | 2.493329994 | 5.630761303 | 0.009323164 | 300 | pseudo |  | RNA, 7SL, cytoplasmic 220, pseudogene |
| SIRPAP1 | 1.506704535 | 2.841602065 | 0.002881048 | 2721 | pseudo |  | signal-regulatory protein alpha pseudogene 1 |
| ZNF341-AS1 | 1.615534149 | 3.064250303 | 0.017937671 | 646 | ncRNA | hsa:101929746 | ZNF341 antisense RNA 1 |
