# Supplementary material for: Whole-Transcriptome Sequence of Degenerative Meniscus Cells Unveiling Diagnostic Markers and Therapeutic Targets for Osteoarthritis
Source: Front Genet. 2021 Oct 15;12:754421. doi: 10.3389/fgene.2021.754421 (PMC8554121; doi:10.3389/fgene.2021.754421)
Supplement: Supplementary file 7 [file DataSheet2.DOCX]

**Supplemental Table 2. Summarized characteristics of healthy and Osteoarthritis patients. Related to Figures 1 to 5, and methods.**

| Average | | |
| --- | --- | --- |
|  | Normal (n=4) | Osteoarthritis (n=15) |
| Age | 38.75  (18-50) | 67.26  (50-80) |
| Male | 75% | 20% |
| Female | 25% | 80% |
| Kellgren-Lawrence grading scores | 0  (0-1) | 3.10  (3-4) |

*Several cases have missing information.
