# Supplementary material for: Whole-Transcriptome Sequence of Degenerative Meniscus Cells Unveiling Diagnostic Markers and Therapeutic Targets for Osteoarthritis
Source: Front Genet. 2021 Oct 15;12:754421. doi: 10.3389/fgene.2021.754421 (PMC8554121; doi:10.3389/fgene.2021.754421)
Supplement: Supplementary file 8 [file DataSheet1.DOCX]

**Supplemental Table 1. Primers for quantitative real-time polymerase chain reaction (qRT-PCR).**

| Gene | Primer sequence (5'-3') |
| --- | --- |
| hsa_circ_0094044-F | TCCTGAACCGGACTGTAACC |
| hsa_circ_0094044-R | AGCTTTGGGGAAAACAGTTT |
| hsa_circ_0005505-F | CACAACGTTCAACCATGCTC |
| hsa_circ_0005505-R | GGTCACCGATGGTCTTGTTT |
| hsa_circ_0018069-F | GAGCATCCGGCAGCACAAAA |
| hsa_circ_0018069-R | TCCGGTAGGCTTGGTCGTTA |
| hsa_circ_0001466-F | CCACCACTGCTCCTCAAC |
| hsa_circ_0001466-R | AAGTGTGGCGATTTTCTCAAG |
| hsa_circ_0000467-F | AGCCCGTAATTGTAACCCCA |
| hsa_circ_0000467-R | TAATGGCTTCTTGCTCGTGT |
| hsa_circ_0000277-F | GAACAGTCTTCAAGGTGGGATC |
| hsa_circ_0000277-R | CCAATCCCAAAGAATCATCTTG |
| hsa-miR-147b-5p | TGGAAACATTTCTGCACAA |
| hsa-miR-184 | TGGACGGAGAACTGATAAG |
| hsa-miR-206 | TGGAATGTAAGGAAGTGTG |
| hsa-miR-146a-5p | TGAGAACTGAATTCCATGG |
| hsa-miR-147b-3p | GTGTGCGGAAATGCTTCT |
| hsa-miR-212-5p | TGGCTCTAGACTGCTTACT |
| lnc-LOC105379771-F | AGCATTAGTTCCATTGGGTCC |
| lnc-LOC105379771-R | AGGCGAGGCTTTCAGACTTTC |
| lnc-LOC105377272-F | GGTTGCTAAGGGTCAGGTTCA |
| lnc-LOC105377272-R | TAGTCTGAGGTTTGTTCCCCA |
| lnc-LOC541472-F | GGGCAGAAAGGGGGAGAATA |
| lnc-LOC541472-R | CCTGCGTCCGTAGTTTCCTT |
| lnc-MT2P1-F | CTCCTGTGCCGCCAGTGAC |
| lnc-MT2P1-R | ACTTGTCCGACGCCCCTTT |
| lnc-DNM1P9-F | GGGTCCCACCTCCTCTATCTG |
| lnc-DNM1P9-R | GGTCGGCGATAGTGCTTCAGT |
| lnc-LOC107985321-F | TCAAGCGATACTCCCCACATA |
| lnc-LOC107985321-R | CTGGAACTCAGGTTTGGCATA |
| lnc-LOC107986251-F | GAGGTGACCAAACTGTGCTTAGAG |
| lnc-LOC107986251-R | CCCCCAAAGCACTTCTCAAAA |
| hsa-SESN3-F | TTTGTGGTCCCTGGAGAAAC |
| hsa-SESN3-R | ATCGAGAGACATCGGCTGTT |
| hsa-TJP2-F | ATGGAAGAGCTGATATGGGAACA |
| hsa-TJP2-R | TGCTGAACTGCAAACGAATGAA |
| hsa-CH25H-F | GGTCATCTTCTCCATCACCACA |
| hsa-CH25H-R | TCCATGTCGAAGAGTAGCAGG |
| hsa-CYP7B1-F | TCTCTTTGCCGCCACCTTAC |
| hsa-CYP7B1-R | AGGCTTTCGCTGATAATCGG |
| hsa-MMP3-F | CTGGACTCCGACACTCTGGA |
| hsa-MMP3-R | CAGGAAAGGTTCTGAAGTGACC |
| hsa-ADAMTS5-F | AATGCACTTCAGCCACCATCA |
| hsa-ADAMTS5-R | TCGTAGGTCTGTCCTGGGAGTTC |
| hsa-ACAN-F | GATGTTCCCTGCAATTACCACCTC |
| hsa-ACAN-R | TGATCTCATACCGGTCCTTCTTCTG |
| hsa-GAPDH-F | GCACCGTCAAGGCTGAGAAC |
| hsa-GAPDH-R | TGGTGAAGACGCCAGTGGA |
| hsa_SERPINB2_F | TCCTGGGTCAAGACTCAAACC |
| hsa_SERPINB2_R | CATCCTGGTATCCCCATCTACAG |
| hsa_PRDM1_F | TAAAGCAACCGAGCACTGAGA |
| hsa_PRDM1_R | ACGGTAGAGGTCCTTTCCTTTG |
| hsa_LCN2_F | GAAGTGTGACTACTGGATCAGGA |
| hsa_LCN2_R | ACCACTCGGACGAGGTAACT |
| hsa_RAB27B_F | AAGGCAGACCTACCAGATCAGAG |
| hsa_RAB27B_R | TTCTCCACACACTGTTCCATTCG |
